# Supplementary material for: How local partnerships to improve urgent and emergency care have impacted delayed transfers of care from hospitals in England: an analysis based on a synthetic control estimation method
Source: BMJ Open. 2022 Feb 7;12(2):e054568. doi: 10.1136/bmjopen-2021-054568 (PMC8823209; doi:10.1136/bmjopen-2021-054568)
Supplement: Supplementary data [file bmjopen-2021-054568supp001.pdf]

Appendix

Table A1: Definition of variables

Variable	Explanation	Frequency	Source
**Dependent**:			
DTOC (days)	Number of days any patient experienced delayed discharge from hospital	Q	NHSE/I
DTOC (log)	Natural logarithm of DTOC(days)	Q	NHSE/I
**Explanatory**:			
UEC Vanguards	1 if one of 29 LA's participating in this Vanguard and it is onwards and after 2015 quarter 3, 0 – otherwise	F	NHSE/I
Care home beds(log)	Natural logarithm of the number of care home beds available in the LA	A	CQC
CA Ratio	Percentage of all population that receives Carer's Allowance	Q	ONS
DLA Ratio(65)	Percentage of older population (65+ years) that receives Disability Living Allowance	Q	ONS
JSA Ratio	Percentage of all population that receives Jobseeker's Allowance	Q	ONS
PC Ratio(65)	Percentage of older population (65+ years) that receives Pension Credit	Q	ONS
Population(log)	Natural logarithm of the LA's population	A	ONS
65+ ratio	Percentage of older people (65+) in the population	A	ONS
House prices(log)	Natural logarithm of average house prices in LA	Q	LandReg
Owned house	Percentage of single occupancy households of older people (65+) that own a house with mortgage, 2011 data	F	ONS
Owned house (outright)	Percentage of single occupancy households of older people (65+) that own a house outright, 2011 data	F	ONS
LA type	Type of Local Authority: Metropolitan, Unitary, London and County	F	Gov
Area	Local Authority's size (m^2)	F	Gov
Rurality	Percentage of people living in rural areas and hub towns, 2011 data	F	ONS
CCGs per LA	Number of CCGs each LA is interacting with	F	Gov
CCG dummy	1 – April 2013 onwards, identifies creation of CCG's, 0 – before April 2013	F	Gov

Note: Q – quarterly, A – annual, F – fixed; NHSE/I – NHS England and Improvement, CQC – Care Quality Commission, ONS – Office for National Statistics, LandReg – HM Land Registry's Price Paid Data, Gov – UK government's online service.

There were no missing values for all data points used throughout the chosen study time period.

Table A2: Synthetic control estimation outcome (DTOC) measures by period:

Period	DTOC days Treated	DTOC days Control (synth)	Difference	Difference in % (to Treated)
1	1569.36	1567.916	-1.444	-0.09201
2	1772.96	1772.207	-0.753	-0.04247
3	1778.4	1779.567	1.167	0.065621
4	1972.96	1969.42	-3.54	-0.17943
5	1918.64	1915.392	-3.248	-0.16929
6	1980.32	1979.21	-1.11	-0.05605
7	2029.24	2026.926	-2.314	-0.11403
8	2274.52	2274.597	0.077	0.003385
9	2164.28	2166.296	2.016	0.093149
10	2137.16	2137.553	0.393	0.018389
11	2062.92	2065.035	2.115	0.102525
12	2028.56	2029.041	0.481	0.023711
13	1996.64	1996.134	-0.506	-0.02534
14	1924.96	1921.385	-3.575	-0.18572
15	1826.32	1821.821	-4.499	-0.24634
16	2063	2056.206	-6.794	-0.32933
17	2061.84	2058.22	-3.62	-0.17557
18	1860.92	1857.615	-3.305	-0.1776
19	1573.04	1572.29	-0.75	-0.04768
**20**	**1701.48**	**1818.133**	**116.653**	**6.855972**
**21**	**1693.04**	**2288.897**	**595.857**	**35.1945**
**22**	**1873.08**	**2313.684**	**440.604**	**23.52297**
**23**	**1871.88**	**2482.475**	**610.595**	**32.61935**
**24**	**2084.72**	**2440.76**	**356.04**	**17.07855**
**25**	**2135**	**2541.185**	**406.185**	**19.02506**
**26**	**1890.96**	**2330.016**	**439.056**	**23.21868**
**27**	**1819**	**2291.485**	**472.485**	**25.97499**
**28**	**1878.28**	**2393.571**	**515.291**	**27.4342**
**29**	**1767.72**	**2232.34**	**464.62**	**26.28357**

Note: Nominal UEC Vanguards start date was 3rd quarter of 2015, quarter 20 in our dataset, results after the start of this Vanguard indicated in bold.

Table A3: Synthetic control estimation predictor balance:

Variable	Treated	Synthetic
JSA ratio	0.039029	0.040455
PC ratio (65+)	0.269154	0.28076
CA ratio	0.011704	0.012353
DLA ratio (65+)	0.098542	0.103666
Care home beds (log)	7.602731	7.35620
Population (log)	12.43287	12.35283
Population 65+ ratio	0.173527	0.160181
Rural or hub (%)	20.8956	12.78465
No. CCGs to LA	4.84	3.924
House prices (£, log)	12.03679	11.99105
Owning single home r. (65+)	0.077565	0.07411
Owning single home outright r. (65+)	0.071337	0.068227
Area(m^2)	79959.32	76051.94

Table A4: Synthetic control post-estimation results:

Post estimation period	Estimates (synth-treated)	p-vals	p-vals standardized
c1 (quarter 20)	-116.653	0.77686	0
c2 (quarter 21)	-595.857	0.38843	0
c3 (quarter 22)	-440.604	0.545455	0
c4 (quarter 23)	-610.595	0.446281	0
c5 (quarter 24)	-356.04	0.719008	0
c6 (quarter 25)	-406.185	0.710744	0
c7 (quarter 26)	-439.056	0.677686	0
c8 (quarter 27)	-472.485	0.628099	0
c9 (quarter 28)	-515.291	0.603306	0
c10 (quarter 29)	-464.62	0.603306	0

Note: post-estimation results using synth_runner command on Stata.
