# Supplementary material for: How local partnerships to improve urgent and emergency care have impacted delayed transfers of care from hospitals in England: an analysis based on a synthetic control estimation method
Source: BMJ Open. 2022 Feb 7;12(2):e054568. doi: 10.1136/bmjopen-2021-054568 (PMC8823209; doi:10.1136/bmjopen-2021-054568)
Supplement: Author's manuscript [file bmjopen-2021-054568.draft_revisions.pdf]

BMJ Open

BMJ Open is committed to open peer review. As part of this commitment we make the peer review history of every article we publish publicly available.

When an article is published we post the peer reviewers' comments and the authors' responses online. We also post the versions of the paper that were used during peer review. These are the versions that the peer review comments apply to.

The versions of the paper that follow are the versions that were submitted during the peer review process. They are not the versions of record or the final published versions. They should not be cited or distributed as the published version of this manuscript.

BMJ Open is an open access journal and the full, final, typeset and author-corrected version of record of the manuscript is available on our site with no access controls, subscription charges or pay-per-view fees (http://bmjopen.bmj.com).

If you have any questions on BMJ Open's open peer review process please email info.bmjopen@bmj.com

BMJ Open

Impact of Urgent and Emergency Care Vanguards on Delayed Transfers of Care in England

Journal:	*BMJ Open*
Manuscript ID	bmjopen-2021-054568
Article Type:	Original research
Date Submitted by the Author:	16-Jun-2021

Complete List of Authors:	Malisauskaite, Gintare; University of Kent, Personal Social Services Research Unit<br>Jones, Karen; University of Kent Personal Social Services Research Unit<br>Allan, Stephen; University of Kent, Personal Social Services Research Unit<br>Roland, Daniel; University of Kent, Personal Social Services Research Unit<br>Birks, Yvonne; University of York, Social Policy Research Unit<br>Baxter, Kate; University of York, Social Policy Research Unit<br>Gridley, Kate; University of York, Social Policy Research Unit

Keywords:	HEALTH ECONOMICS, Health policy < HEALTH SERVICES ADMINISTRATION & MANAGEMENT, Organisation of health services < HEALTH SERVICES ADMINISTRATION & MANAGEMENT

SCHOLARONE™
Manuscripts

I, the Submitting Author has the right to grant and does grant on behalf of all authors of the Work (as defined in the below author licence), an exclusive licence and/or a non-exclusive licence for contributions from authors who are: i) UK Crown employees; ii) where BMJ has agreed a CC-BY licence shall apply, and/or iii) in accordance with the terms applicable for US Federal Government officers or employees acting as part of their official duties; on a worldwide, perpetual, irrevocable, royalty-free basis to BMJ Publishing Group Ltd ("BMJ") its licensees and where the relevant Journal is co-owned by BMJ to the co-owners of the Journal, to publish the Work in this journal and any other BMJ products and to exploit all rights, as set out in our licence.

The Submitting Author accepts and understands that any supply made under these terms is made by BMJ to the Submitting Author unless you are acting as an employee on behalf of your employer or a postgraduate student of an affiliated institution which is paying any applicable article publishing charge ("APC") for Open Access articles. Where the Submitting Author wishes to make the Work available on an Open Access basis (and intends to pay the relevant APC), the terms of reuse of such Open Access shall be governed by a Creative Commons licence – details of these licences and which Creative Commons licence will apply to this Work are set out in our licence referred to above.

Other than as permitted in any relevant BMJ Author's Self Archiving Policies, I confirm this Work has not been accepted for publication elsewhere, is not being considered for publication elsewhere and does not duplicate material already published. I confirm all authors consent to publication of this Work and authorise the granting of this licence.

For peer review only

Impact of Urgent and Emergency Care Vanguards on Delayed Transfers of Care in England

Gintare Malisauskaite*[1], Karen Jones[1], Stephen Allan[1], Daniel Roland[1], Yvonne Birks[2], Kate Baxter[2], Kate Gridley[2]

*Correspondence: G.Malisauskaite@kent.ac.uk

Personal Social Services Research Unit (PSSRU), University of Kent, Office CC228, Cornwallis Central, Canterbury, CT2 7NF, UK

Word count: 3182

[1] Personal Social Services Research Unit (PSSRU), School of Social Policy, Sociology and Social Research (SSPSSR), University of Kent, UK

[2] Social Policy Research Unit (SPRU), Department of Social Policy and Social Work, University of York, UK

For peer review only

Abstract

Objectives: One of the aims of Urgent and Emergency Care (UEC) Vanguards was integration of health and social care sectors, the success of which is expected to be reflected in days of Delayed Transfers of Care (DTOC). Consequently, we assessed the relationship between UEC Vanguards and DTOC at Local Authority (LA) level in England.

Design: Using a synthetic control estimation method 29 UEC partner LAs were averaged into a single 'treated' unit and compared with a unit created using data from non-UEC English LAs to estimate the impact of UEC Vanguards on DTOC days. Fixed effects panel regressions were used to quantify the effect size.

Setting: 150 Local Authorities in England (excluding city of London and Isles of Scilly); 29 LAs were partners in Urgent and Emergency Care (UEC) Vanguards between August 2015 and March 2018.

Primary outcome measure: Quarterly data on days of Delayed Transfers of Care (DTOC) at LA level for the period 2010 to 2017.

Results: Synthetic control estimation showed a large difference in DTOC days between UEC and non-UEC LAs, with on average 21% lower DTOC per quarter (507 DTOC days per quarter). Fixed effect panel regressions found DTOC rates were lower by 40.5% (99% confidence interval: 16.7-64.3%) in UEC partner LAs after the start of the Vanguards programme. We found no indication of UEC participant sites having lower DTOC rates prior to initiation of Vanguards.

Conclusions:

The evidence indicates a sizeable statistically significant impact of UEC Vanguards on DTOC, however, more research is required to explain the underlying reasons for this relationship.

Keywords:

For peer review only

Delayed Transfers of Care, Urgent and Emergency Care Vanguards, integration policy

Article Summary:

- The long span of DTOC data used from all English Local Authorities created a comprehensive picture of how UEC Vanguards affected DTOC rates;
- The synthetic control method allowed for a comparison between UEC and non-UEC LAs to be drawn allowing observation of how UEC partner LAs would have been likely to perform in DTOC days in the absence of UEC Vanguards;
- The data did not permit the identification and estimation of the impact of different initiatives associated to the UEC Vanguards or account for differences in breadth and reach of UEC Vanguards within the partner LAs;
- The design did not permit a clear explanation of the mechanisms by which UEC Vanguards influenced reduction in DTOC days.

Funding

This study was funded by the National Institute for Health Research School for Social Care Research, grant number C088/CM/UKJB-P116. The views expressed in this independent research publication are those of the authors and not necessarily those of the NIHR SSCR, the National Institute for Health Research or the Department of Health and Social Care.

Competing interests

The authors declare that they have no competing interests.

Data Statement

Technical appendix, statistical code, and dataset available from the corresponding author on reasonable request.

For peer review only

Background

Delayed Transfers of Care (DTOC) is a term used to describe situations where patients are medically fit to be discharged from hospital to home or further care settings but the process is delayed (1, 2). DTOC has attracted increased attention from policy makers alongside health and social care professionals in England (3), due to increased rates in recent years; there is an estimated £820 million annual cost for the population aged 65+ (2015-2016 estimate) (4). DTOC is also associated with decreased subsequent participation in activities of daily living, frailty, aging, high comorbidity, cognitive impairment, and dependency (5-11).

Attempts to address the costs associated with DTOC have inspired a number of innovative policy approaches to integrating social and health care, including the Better Care Fund (12), Integrated Care Pioneers (13) and more recently the New Models of Care – Vanguards (14). Vanguards set out to help improve integration of services with five different approaches (14):

- *Acute care collaboration* – linking local hospitals to improve clinical and financial viability.
- *Urgent and emergency care* – improving coordination of services and reducing pressure on A&E departments.
- *Enhanced health in care homes* – improving and integrating health, care and rehabilitation services for older people in care homes.
- *Multi-speciality community providers* – moving specialist care into the community from hospitals.
- *Integrated primary and acute care systems* – joining up GP, hospitals, community and mental health services.

This paper examines the relationship between Urgent and Emergency Care (UEC) Vanguards and DTOC rates specifically, since discharge planning from acute care was identified as one of the challenges by the UEC Vanguards (15). DTOC may be directly affected

For peer review only

by the effectiveness of communication between health and social care providers. As such, DTOC rates may be a useful proxy for certain aspects of integration. For example, DTOC has previously been used as a criterion for evaluating the success of health and social care integration policy initiatives (16).

Eight UEC Vanguards were announced to take effect in July-August 2015 with a planned end date of March 2018, however a substantial number of Vanguards managed to provide enhanced services beyond that date (17). UEC models of care largely involved integrated urgent care accessed through NHS 111 alongside developing a Channel Shift modelling tool (18) to facilitate service planning and design. The latter, especially, was aimed at further integration and cooperation between health, social and community care services, and included planning for discharge from the hospital from the point of admission (14-15). For this reason we determined there to be sufficient rationale for testing any relationship between Vanguards and delayed discharges from hospitals.

To date, research related to DTOC is somewhat scarce. Most research considers challenges related to the discharge of older people: appropriate future support, suitable discharge destinations, and how policies are put into practice (19-22). Waiting for post-hospital care packages accounts for a large proportion of reasons for delays (23) and lack of social care supply is considered a major part of the explanation behind DTOC (24). However, whilst delays for which social care is responsible have increased substantially, so have NHS delays (25, 26). Indeed, the NHS is continuously considered responsible for the majority of delays, with internal hospital issues with planning, documentation and transport being cited as explaining increases in DTOC (27) alongside concerns over the integration of services more generally (28, 29). To contribute to this literature and as part of a wider study examining the role of social care in DTOC (30) we examined the link between UEC Vanguards and DTOC rates.

For peer review only

Methods

Data were collected for 150 English Local Authorities (LAs) for the time period between 2010 quarter 4 and 2017 quarter 4 (150 LAs, 29 quarters, 4,350 observations). City of London and the Isles of Scilly were excluded due to stark differences in size in comparison to other LAs. The analysis was carried out at quarterly LA level; the time frame chosen based on the availability of DTOC data at the time of analysis. The eight UEC Vanguards took effect in August 2015 (calendar year quarter 3) and included 29 Local Authorities as partners (14). The start date of UEC Vanguards was nominal, but without more precise information on exact timings of when the programmes took effect in different locations or when it was likely to expect any impact on outcomes, we used this nominal start date as the start of the 'treatment' in our analyses.

The primary chosen method of analysis was synthetic control estimations (31, 32). This method creates a control unit that matches the main characteristics of the treated unit so that it has a similar outcome trajectory prior to the treatment. This then allows observation of how the 'untreated' control unit would have performed in the time following the start of the treatment, providing a comparison with the treated unit. In this case the 'treatment' was a UEC Vanguard programme and by the 'treated unit' we refer to derived averages of the outcome measure and control variables (33) of all 29 LA partners of UEC Vanguards partner sites, i.e. the 29 LAs at each quarter were used in the sample to create a single treated unit over time. The control unit was created using the remaining LAs in England (i.e. non-UEC Vanguards) by estimating different weights, chosen automatically by the synthetic control estimation algorithm, for LAs to account for changes in confounding variables over time as well as across LAs. Further, we controlled for the outcome variable at each quarter prior to the treatment to achieve close tracking between the two units over the period prior to the treatment. This allowed for a credible

For peer review only - http://bmjopen.bmj.com/site/about/guidelines.xhtml

prediction of the counterfactual i.e. what would have happened in the absence of the Vanguards programme in the UEC partner LAs. DTOC trends not being parallel prior to Vanguards makes synthetic control estimation a preferred method for evaluation since it does not require parallel trends assumption to hold.

The outcome measure used for synthetic control estimations was the number of DTOC days (including all delayed discharge days). The analysis controlled for the following factors associated with LA-level DTOC: (i) demographics and level of need (carer's allowance, disability living allowance, total population in LA, and percentage of population above 65 years old), (ii) LA structure (type, size in square metres, percentage of people living in rural areas, the number of Clinical Commissioning Groups (CCGs) each LA is in partnership with, and care home bed supply), and (iii) economic variables (jobseeker's allowance, pension credit, house prices, percentage of older people home ownership. A similar set of control variables were selected by Roland et al. (34) based on findings from the literature (5-9, 19-23) concerning confounders of delayed discharges from hospitals and data availability. Further details of the data are available in Appendix Table A1.

Since synthetic control estimation does not produce a single estimate of the effect size of the Vanguards programme on DTOC, we also estimated fixed effects panel regressions. This provided a single estimate of the treatment size for reference purposes. The model specification used was:

$$DTOC_{it} = \alpha_i + V_{it}\beta_1 + C_{it}\beta_2 + u_{it} \quad (1)$$

Where the dependent variable $DTOC_{it}$ is expressed as the natural logarithm of the number of DTOC days, to mitigate the potential effect of large outliers and data skewness in regression results. Subscript i indicates a Local Authority, t time (in quarters) and α is a LA dependent intercept. Vanguard partners were identified using the dummy variable V_{it} (1 = after programme start, 2015 quarter 3, 0 = before or not Vanguard partner), and β_1 is the coefficient

of interest for the analysis, showing the average effect of being a partner in the UEC Vanguards on DTOC. Finally, C_{it} is a vector of the same control variables used in the synthetic control estimation and year dummies for each year since 2010, with β_2 being a vector of coefficients associated with them, representing the effects they have on DTOC rate.

The equation (1) was estimated using fixed effects panel regressions (35). A Hausman test found that a fixed effects model was preferred over a random effects model (Hausman test statistic of 63.44, significant at 1%). Some variables were dropped during the estimation process due to the invariant nature of some data (e.g. LA type and size etc.). All regressions used cluster-robust standard errors centred on LAs, to account for potential heteroscedasticity and unobservable characteristics which could make LA level clusters more similar.

Finally, given the potential for selection bias into UEC vanguards, as robustness checks we checked if there was any evidence of significant differences in DTOC rates between UEC and non-UEC LAs by a) assuming that the UEC programme was in existence across the whole period of analysis and b) using data from prior to the start of the programme. These robustness checks were estimated using Ordinary Least Squares (OLS) and random effects (RE) given UEC vanguard partners were assumed fixed over time.

Patient and Public Involvement statement

Both public and practice stakeholders were involved in the project as part of the steering committee. They helped to shape initial study design and participated in the discussion of initial and final findings alongside dissemination. Further stakeholder engagement through dissemination at practice facing events looking at findings further shaped the presentation of early findings.

Results

[Table 1]

For peer review only

[Table 2]

Descriptive statistics

Table 1 presents descriptive statistics from the last quarter of 2010 to the last quarter of 2017. The average number of DTOC days was 2,755 per English LA per quarter. 6.7% of the sample observations were UEC Vanguard partner sites. Table 2 provides more detail on outcome and control variables based on participation in UEC Vanguard programme presenting mean values before and after the start of UEC Vanguards. Before and after means for control variables appear similar for both UEC and non-UEC partner sites, the main noteworthy difference being the sizeable increase in DTOC days after the start of the Vanguards in non-UEC LAs.

[Figure 1]

Figure 1 compares average number of DTOC days (ratio to LA population) per quarter for UEC partner LAs compared to those non-UEC LAs. The increase in DTOC days after the start of the UEC programme, obvious for the non-UEC LAs, was hardly visible for LAs in UEC Vanguards. However, since the trends prior to the Vanguards seem non-parallel, robustness checks were employed to check for any indication that selection into the UEC Vanguards programme was based on DTOC rates.

Main results

Figure 2 depicts the predictions of synthetic control estimations. The figure shows that from the beginning of the UEC Vanguards programme, partner sites had consistently lower than average DTOC rates than would have been predicted without the Vanguards. This difference was sizeable, with on average 507 fewer DTOC days per quarter per LA, or around 21% less (estimation outcome matrix, showing actual average days of DTOC for UEC partner LAs and predicted synthetic control unit days of DTOC, is provided in Table A2 in the Appendix). The synthetic control tracking the data prior to UEC programme indicates a good fit for the model

For peer review only

(the predictor balances between averaged treated unit and synthetic control unit are available in Table A3 in Appendix). The sizeable dip in DTOC days just prior to the start of UEC Vanguards of, on average, 300 delayed days less per UEC Vanguard participating LA compared to the previous quarter, suggests that some form of preparation with regard to integration and DTOC could have taken place in the participating sites.

[Figure 2]

Fixed effects panel regression

The first column of Table 3 reports the full results from the fixed effects panel regression estimation and shows UEC Vanguards are associated with 40.5% lower DTOC rates at 1% significance level (99% confidence interval: 16.7 – 64.3%). It was a preferred estimation for finding the coefficient size associated with UEC Vanguards impact, since when controlling for LA level characteristics Hausman test revealed systematic differences between fixed effects and random effects models and fixed effects model has an advantage of controlling for any other unobservable LA level characteristics that are fixed in time.

[Table 3]

Robustness checks

One of the possible interpretations for the observed results may be that sites participating in UEC Vanguards could have been selected based on better DTOC rates. There was no publicly available information on specific selection criteria but testing for statistical differences between DTOC rates was carried out by running the model using two modifications:

a) without identifying the start of the UEC Vanguard, checking for an overall difference between UEC participating sites and others over the entire period of time of data;

b) using only data prior to 2015, the start of the initiative.

The findings (Table 3, columns a) and b) respectively) showed there was no statistically significant relationship between UEC Vanguards and DTOC in these alternative specifications.

For peer review only

This suggests that the difference in DTOC rates between UEC and non-UEC partner LAs only becomes significant after the start of UEC programme.

Discussion

Main findings

Our findings suggest that LAs that were part of UEC Vanguards had significantly lower average DTOC than non-UEC sites after the start of the programme. Overall DTOC rates rose substantially in the second quarter of 2015 until the end of 2016, potentially explained by severe cuts to the funding of social care (36); however, they rose significantly less, if at all, in UEC Vanguard sites. We found no evidence suggesting that UEC Vanguard sites had lower DTOC rates prior to becoming Vanguards but are unable to rule out the possibility that they might have been in a better position to reduce DTOC due to other as yet unidentified factors, e.g. other health care programmes already in place in the UEC partner LAs. It is difficult to pinpoint the reasons why UEC Vanguards influenced DTOC rates. One potential explanation from policies and initiatives associated with being in a Vanguard is the Channel Shift modelling tool-kit, which supports integration of services, communication and cooperation between hospital and community based services (18). Channel Shift interventions include planning discharge from time of admission, discharging for further assessment ('discharge to assess') and rapid response services, yet we could not formally test if this indeed is the main reason for the effect found.

There is limited previous research that has looked at the relationship between New Models of Care – Vanguards and DTOC rates. One study reported that Vanguards are associated with a small reduction in hospital admissions (37). This highlights important implications for the National Health Service and social care, alongside individuals discharged from hospital. The move towards greater integration of services is not a new idea, and the importance of local

For peer review only

collaboration has been consistently stressed (38). However, it is difficult to isolate the impact of the UEC Vanguards from other Government integration policies, together with the austerity climate facing the care sector. This is particularly important with regard to potential changes the COVID-19 response has led to, including hospital discharge service, which is expected to influence the further development of discharge to assess and integration of health and social care services (39). Although New Care Models were discontinued well before the start of the global pandemic, assumptions were made that practices adopted during the time of New Care Models activity period would continue to be used (17). Alongside our findings, this indicates that the Vanguards programme should be of interest to policy makers in terms of lessons learned for dealing with discharges during and following the COVID-19 pandemic.

Limitations

The synthetic control estimation suggests an element of causality between UEC participation and DTOC rates. However, a number of limitations need to be acknowledged. Firstly, we could not account for selection criteria into the Vanguards, different initiatives within UEC Vanguards and the impact of other active health policies potentially influencing the results during the Vanguards' activity period. Such information could further inform analysis and provide further context for this finding. Further work on specific policies used within UEC Vanguard sites, including qualitative analysis and more detailed examination of specific UEC Vanguards' mechanisms of action could help untangle potential reasons for the association between UEC Vanguards and DTOC.

Secondly, this analysis does not account for different timeframes in which UEC Vanguards took effect in different locations, differences within the eight UEC Vanguards, or possible different levels of exposure to UEC Vanguard influence in each LA. We anticipate there was some variation in associations between different UEC Vanguards and outcomes. However, this approach should be sufficient to show the average effect of the UEC Vanguards programme.

For peer review only

The identified limitations could be further routes of enquiry, even if achieving precision in quantifying health care programmes is unlikely.

A potential solution to refining the analysis would be to use individual level data to conduct a similar analysis. This could allow more precise estimation of the effect size as case-specific confounders could be taken into account, which is not possible at an aggregated level.

Overall, the estimation methods adopted revealed a strong association between the UEC Vanguard and a reduction of DTOC, which advocates the success of this integration programme and should encourage further research to reveal which specific aspects of this initiative were particularly beneficial, which could guide further policy decisions.

For peer review only

Abbreviations

UEC: Urgent and emergency care vanguard;

DTOC: delayed transfers of care;

LA: Local Authority;

CCG: Clinical Commissioning Group;

FE: Fixed effects panel regression model.

OLS: Ordinary least squares;

Author Contributions

GM conceived the statistical methodology, performed the statistical analysis and drafted the manuscript; KJ contributed to the conception, study design and the final writing of this paper; SA contributed to the study design, statistical methodology and the final writing of this paper; DR contributed to the statistical methodology and the final writing of this paper; YB critically revised the draft and contributed to the final writing of this paper; KB critically revised the

draft and contributed to the final writing of this paper; KG critically revised the draft and contributed to the final writing of this paper.

References

1. NHS England. Delayed Transfers of Care. Leeds: NHS England; 2011 [updated 2019]. https://www.england.nhs.uk/statistics/statistical-work-areas/delayed-transfers-of-care/. Accessed Oct 2019.
2. The Kings Fund. Delayed transfers of care: a quick guide. London: The Kings Fund; 2018. https://www.kingsfund.org.uk/publications/delayed-transfers-care-quick-guide. Accessed Nov 2020.
3. NHS Providers. The rise of delayed transfers of care. London: NHS Providers; 2017. https://nhsproviders.org/the-rise-of-delayed-transfers-of-care. Accessed Oct 2019.
4. National Audit Office. Discharging older patients from hospitals. London: National Audit Office; 2016. https://www.nao.org.uk/wp-content/uploads/2015/12/Discharging-older-patients-from-hospital.pdf. Accessed Apr 2019.
5. Bo M, Fonte G, Pivaro F, et al. Prevalence of and factors associated with prolonged length of stay in older hospitalized medical patients. *Geriatr Gerontol Int.* 2016;16(3):314-321.
6. Challis D, Hughes J, Xie C, Jolley D. An examination of factors influencing delayed discharge of older people from hospital. *Int J Geriatr Psychiatry.* 2014;29(2):160-168.
7. Costa AP, Hirdes JP. Clinical characteristics and service needs of alternate-level-of-care patients waiting for long-term care in Ontario hospitals. *Healthc Policy.* 2010;6(1):32-46.
8. Costa AP, Poss JW, Pierce T, Hirdes JP. Acute care inpatients with long-term delayed-discharge: Evidence from a Canadian health region. *BMC Health Serv Res.* 2012;12:172-182.
9. McClaran J, Tover R, Duarte E. Long hospital stays and need for alternate level of care at discharge. Does family make a difference for elderly patients? *Can Fam Physician.* 1996;42:449-461.
10. Rojas-García A, Turner S, Pizzo E, Hudson E, Thomas J, Raine R. Impact and experiences of delayed discharge: A mixed-studies systematic review. *Health Expect*. 2018;21(1):41-56.
11. Bai AD, Dai C, Srivastava S, Smith CA, Gill SS. Risk factors, costs and complications of delayed hospital discharge from internal medicine wards at a Canadian academic medical centre: retrospective cohort study. *BMC Health Serv Res.* 2019; doi: 10.1186/s12913-019-4760-3.
12. NHS England. Better Care Fund. London: NHS England; 2013. https://www.england.nhs.uk/ourwork/part-rel/transformation-fund/bcf-plan/. Accessed Oct 2019.

13. NHS England. Integrated Care Pioneers. London: NHS England; 2014. https://www.england.nhs.uk/new-care-models/integrated-care-pioneers/. Accessed Oct 2019.

14. NHS England. New Care Models: Vanguards – developing blueprint for the future of NHS and care services. London: NHS England; 2016. https://www.england.nhs.uk/wp-content/uploads/2015/11/new_care_models.pdf. Accessed Mar 2019.

15. NHS England. New Care Models: supporting the vanguards Urgent and Emergency Care (UEC) vanguards: summary. London: NHS England; 2015. https://www.england.nhs.uk/wp-content/uploads/2015/12/uec-support-package-summary.pdf. Accessed Oct 2019.

16. Forder J, Caiels J, Harlock J, Wistow G, Malisauskaite G, Peters M, Marczak J, D'Amico F, Fernandez JL, Fitzpatrick R, Jones K. A system-level evaluation of the Better Care Fund: Final report. Personal Social Services Research Unit; 2018. https://www.pssru.ac.uk/pub/5424.pdf. Accessed Oct 2019.

17. National Health Executive. Vanguards: Stevens pledges 10-year plan, original aims failed due to trust deficits. 2018. https://www.nationalhealthexecutive.com/Health-Care-News/vanguards-stevens-pledges-10-year-plan-original-aims-failed-due-to-trust-deficits

18. NHS England. Urgent and emergency care channel shift model. London: NHS England; 2015. https://www.england.nhs.uk/urgent-emergency-care/about-uec/uec-channel-shift/. Accessed Oct 2019.

19. Coffey A. Discharging older people from hospital to home: what do we know? Int. *J. Older People Nurs.* 2006;1:141-150.

20. Glasby J, Littlechild R, Pryce K. All dressed up and nowhere to go? Delayed hospital discharges and older people. *J. Health Serv. Res. Pol.* 2006;11(1):52-58.

21. Swinkels A, Mitchell T. Delayed transfer from hospital to community settings: the older person's perspective. *Health Soc. Care Community.* 2008;17(1):45-53.

22. Shaw J, Kontos P, Martin W, Victor C. The inter-relation between policy and practice for transitions from hospital to home: an ethnographic case study in England's National Health Service. *BMC Health Serv Res.* 2014;P111. doi:10.1186/1472-6963-14-S2-P111.

23. The Nuffield Trust. Delayed transfers of care. We look at delayed transfers of care for patients following NHS treatment. 2021. The Nuffield Trust. https://www.nuffieldtrust.org.uk/resource/delayed-transfers-of-care. Accessed May 2021.

24. Gaughan J, Gravelle H, Siciliani L. Testing the bed-blocking hypothesis: does nursing and care home supply reduce delayed hospital discharges? J.*Health Econ.* 2015;24(1):32-44.

25. Maguire D. Delayed transfers of care: a target that misses the mark? 2018. The King's Fund. Blog. https://www.kingsfund.org.uk/blog/2018/01/delayed-transfers-care-target-misses-mark. Accessed May 2021.

26. Bottery S. Stopping the blame game around delayed transfers of care. 2017. King's Fund. Blog. https://www.kingsfund.org.uk/blog/2017/11/stopping-blame-game-delayed-transfers-care. Accessed May 2021.

For peer review only

27. Edwards N. What's behind delayed transfers of care? 2017. The Nuffield Trust. Briefing. https://www.nuffieldtrust.org.uk/resource/what-s-behind-delayed-transfers-of-care. Accessed May 2021.

28. Fernandez J, McGuire A, Raikou M. Hospital coordination and integration with social care in England: The effect on post-operative length of stay. *J.Health Econ.* 2018;61:233-243.

29. Goddard M, Mason A. Integrated care: A pill for all ills? *Int J Health Policy Manag*. 2017;6(1):1-3.

30. Jones K, Allan S, Malisauskaite G, Birks Y, Baxter K, Gridley K, Roland D. The influence of social care on delayed transfers of care (DTOC) among older people: Final report. Personal Social Services Research Unit. 2020. https://kar.kent.ac.uk/id/eprint/77826. [In press].

31. Abadie A, Diamond A, Hainmueller J. Synthetic Control Methods for Comparative Case Studies: Estimating the Effect of California's Tobacco Control Program. *J. Am. Stat. Assoc*. 2010;105(490):493-505.

32. Abadie A, Diamond A, Hainmueller J. Comparative Politics and the Synthetic Control Method. *Am. J. Political Sci.* 2015;59(2):495-510.

33. Kreif N, Grieve R, Hangartner D, Turner A J, Nikolova S, Sutton M. Examination of the Synthetic Control Method for Evaluating Health Policies with Multiple Treated Units. *Health Econ*. 2015; 25(12):1514-1528.

34. Roland D, Malisauskaite G, Jones K, Birks Y, Baxter K, Gridley K, Allan S. Identifying unexplained DTOC at Local Authority level. 2020. [Forthcoming].

35. Wooldridge J M. Econometric analysis of cross section and panel data. 2nd ed. Cambridge: The MIT Press; 2010.

36. Bate A. Delayed transfers of care in the NHS. Briefing paper Nr. 7415, London: House of Commons Library; 2017. https://commonslibrary.parliament.uk/research-briefings/cbp-7415/. Accessed Oct 2019.

37. Morciano M, Checkland K, Billings J, Coleman A, Stokes J, Tallack C, Sutton M. New integrated care models in England associated with small reduction in hospital admissions in longer-term: a difference-in-differences analysis. *Health Policy*. 2020;124(8):826-833.

38. NHS England. Why not home? Why not today? Behind every delayed transfer of care from hospital to home, there is a person, in the wrong place at the wrong time. Monthly Delayed Transfers of Care Situation Report. London: NHS England; 2018. https://www.england.nhs.uk/statistics/wp-content/uploads/sites/2/2018/11/Monthly-Delayed-Transfers-of-Care-Situation-Report-Principles-Definitions-and-Guidance.pdf. Accessed Jun 2020.

39. HM Government. Hospital Discharge Service: Policy and Operating Model. London: Department of Health and Social Care; 2020. https://assets.publishing.service.gov.uk/government/uploads/system/uploads/attachment_data/file/912199/Hospital_Discharge_Policy_1.pdf. Accessed Nov 2020.

[Appendix]

For peer review only

Tables and Figures

Table 1: Descriptive statistics

Variable	Mean	SD	Min/Max
**Dependent variables:**			
DTOC days	2755.394	3124.008	0/26733
DTOC(log)	7.438	0.986	0/10.194
**Explanatory variables:**			
UEC Vanguard	0.067	0.249	0/1
JSA ratio	0.026	0.016	0.001/0.09
PC ratio (65+)	0.235	0.096	0.062/0.691
CA ratio	0.011	0.004	0.004/0.027
DLA ratio (65+)	0.087	0.040	0.025/0.257
Care home beds (log)	7.553	0.825	5.451/9.461
Population (log)	12.598	0.606	10.519/14.257
Population 65+ ratio	0.166	0.043	0.06/0.286
Rural or hub (%)	17.507	24.468	0/100
No. CCGs to LA	5.660	3.480	1/21
House prices (£, log)	12.343	0.519	11.443/14.62
Owning house ratio (65+)	0.075	0.022	0.013/0.126
Owning house outright ratio (65+)	0.069	0.021	0.011/0.115
Area(m^2)	86839.430	150126.3	1213/803761
CCG dummy	0.655	0.475	0/1
LA type:			
Metropolitan	0.240	0.427	0/1
London	0.213	0.410	0/1
County	0.180	0.384	0/1
Year:			
2010	0.034	0.182	0/1
2011	0.138	0.345	0/1
2012	0.138	0.345	0/1
2013	0.138	0.345	0/1
2014	0.138	0.345	0/1
2015	0.138	0.345	0/1
2016	0.138	0.345	0/1
2017	0.138	0.345	0/1
No of obs.		4,350	

For peer review only

Table 2: Means of outcome and control variables by UEC Vanguard participation, before and after

	Non-UEC		UEC	
	Before	After	Before	After
DTOC days	2510.955	3690.847	2130.523	2233.072
DTOC(log)	7.344334	7.758407	7.255489	7.297711
JSA ratio	0.031272	0.012509	0.038485	0.016309
PC ratio (65+)	0.254087	0.190102	0.263469	0.19152
CA ratio	0.010333	0.012434	0.011437	0.014433
DLA ratio (65+)	0.087383	0.080628	0.097996	0.090038
Care home beds (log)	7.521928	7.535296	7.666286	7.666745
Population (log)	12.60945	12.63799	12.5038	12.52737
Population 65+ ratio	0.161205	0.168712	0.174282	0.183642
House prices (£, log)	12.34778	12.52908	12.03327	12.17675
Rural or hub (%)	16.31793		22.46746	
No. CCGs to LA	5.752066		5.275862	
Owning house ratio (65+)	0.074886		0.077415	
Owning house outright ratio (65+)	0.068836		0.071335	
Area(m^2)	83452.44		100971.3	
No of obs.	2,420	1,210	551	290

Note: Before – 2010 q4 to 2015 q2, 19 quarters; After – 2015 q3 to 2017 q4, 10 quarters.

Table 3: Regression results with DTOC (log) as dependent variable

Variable	FE	RE		OLS	
		a)	b)	a)	b)
UEC Vanguard	-0.405***	-0.044	0.147	-0.047	0.132
	[0.091]	[0.086]	[0.096]	[0.079]	[0.095]
JSA ratio	-4.051	-3.992	-1.6335	4.135	8.027
	[4.851]	[4.142]	[3.110]	[4.405]	[5.333]
PC ratio (65+)	3.188	2.576*	1.025	1.197	0.842
	[2.310]	[1.358]	[1.288]	[0.838]	[0.969]
CA ratio	51.545	-5.468	15.196	-39.645**	-47.167*
	[45.639]	[25.086]	[26.198]	[17.904]	[25.523]
DLA ratio (65+)	0.865	-2.839	-1.174	0.229	0.915
	[5.673]	[2.219]	[2.445]	[1.587]	[1.738]
Care home beds (log)	-0.483	-0.250	-0.231	-0.164	-0.234
	[0.351]	[0.215]	[0.226]	[0.159]	[0.194]
Population (log)	2.103	1.528***	1.599	1.384***	1.513***
	[2.074]	[0.272]	[0.263]	[0.197]	[0.237]
Population 65+ ratio	20.096**	11.455**	12.162	1.977	2.578
	[9.470]	[5.692]	[5.754]	[3.214]	[4.578]
House prices (£, log)	0.400**	0.180	0.353	0.113	0.213
	[0.199]	[0.124]	[0.111]	[0.129]	[0.153]
CCG dummy	0.004	0.034	0.022	0.090**	0.110
	[0.038]	[0.034]	[0.033]	[0.036]	[0.040]
Rurality (%)	-	-0.010	-0.011	-0.005	-0.006
		[0.006]	[0.008]	[0.005]	[0.008]
No. CCGs to LA	-	-0.031**	-0.038	-0.032***	-0.033**
		[0.014]	[0.015]	[0.012]	[0.014]
House ownership ratio (65+)	-	-126.340**	-179.023	-76.799*	-129.735**
		[53.754]	[62.096]	[40.174]	[50.759]
House ownership outright ratio (65+)	-	127.150**	178.562	84.682**	140.397***
		[53.566]	[60.882]	[41.060]	[50.909]
Area(m^2)	-	0.000	-0.000	0.000	0.000
		[0.000]	[0.000]	[0.000]	[0.000]
LA Type:					
Metropolitan	-	-0.322**	-0.47	-0.203*	-0.343***
		[0.147]	[0.142]	[0.103]	[0.122]
London	-	-0.625***	-0.646	-0.621***	-0.664***
		[0.162]	[0.158]	[0.119]	[0.140]
County	-	0.121	0.096	0.227	0.208
		[0.202]	[0.210]	[0.182]	[0.200]
Cons.	0.004	-12.853***	-15.933***	-9.837***	-12.485***
	[0.038]	[2.734]	[2.420]	[2.153]	[2.594]
No. of obs.	4,350	4,350	2550	4,350	2550
No. of groups	150	150		150	

Note: Robust clustered standard errors in square brackets underneath the coefficients. Variables omitted during the FE estimation process due to fixed effects nature of the model: LAs' rural or hub (%), no. CCGs to LA, owning house ratio (65+), owning house outright ratio (65+), area(m^2), LA type; more details of variable construction available in the Appendix; model also includes year indicators between 2010-2017.
Robustness checks in RE and OLS columns: a) UEC Vanguard partner LAs are identified with a binary variable across the whole period of analysis; b) UEC Vanguard partner LAs are identified with a binary variable only using sample prior to 2015. FE results not available for these specifications due to treatment being fixed across time in the estimations.
*** - p<1%, ** - p<5%, * - p<10%

For peer review only

Figure 1: Average DTOC days over time

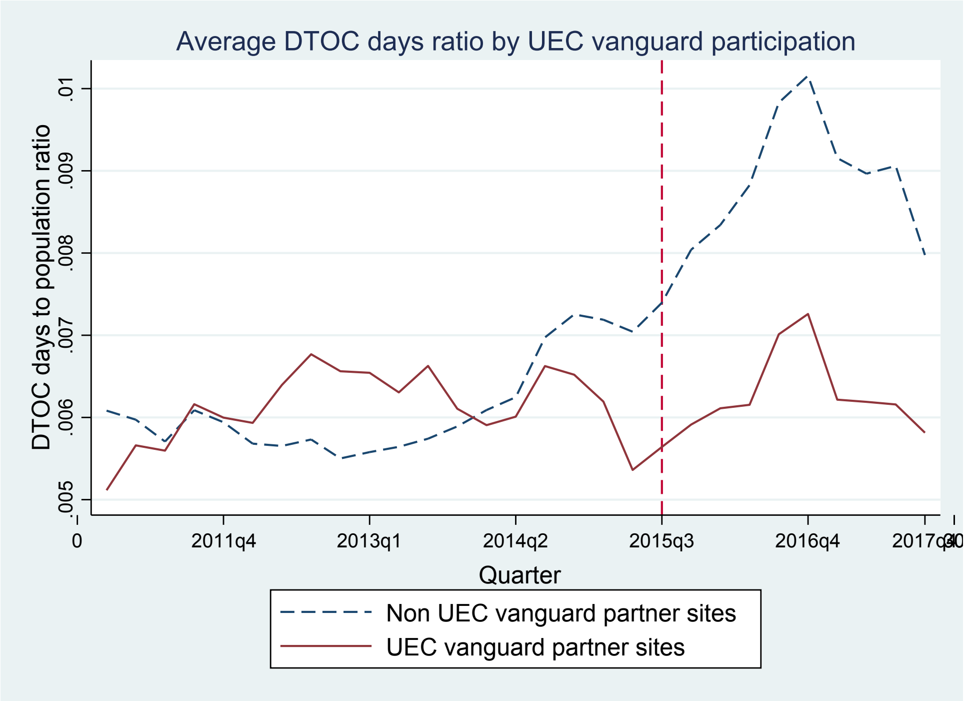

Note: UEC Vanguard partner sites include 29 LA's (12).

For peer review only

Figure 2: Synthetic control estimations

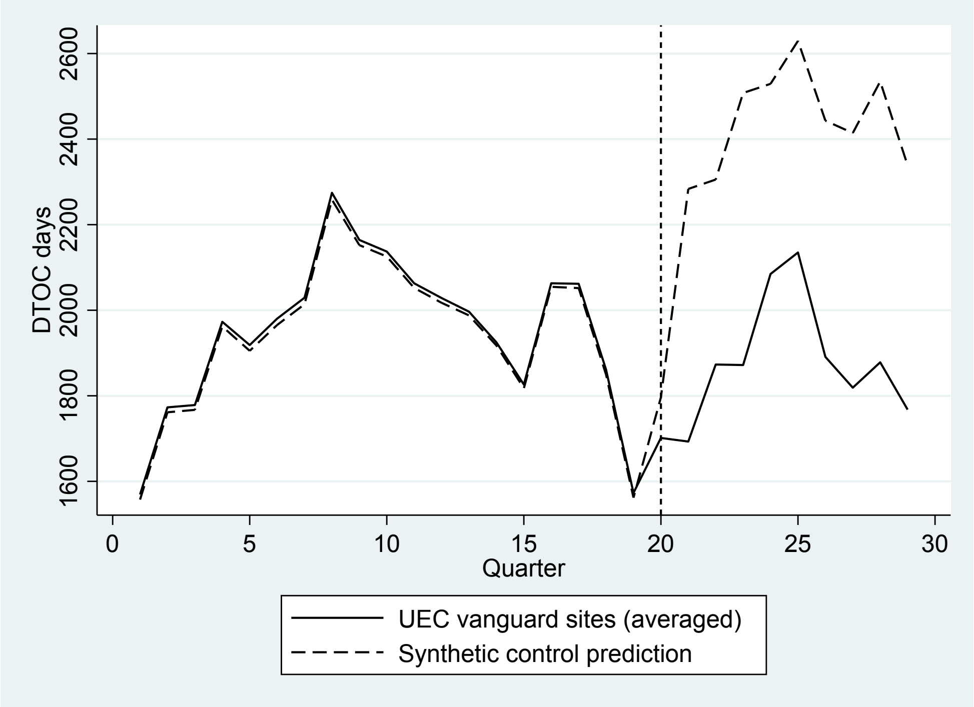

For peer review only

Appendix

Table A1: Definition of variables

Variable	Explanation	Frequency	Source
**Dependent**:			
DTOC (days)	Number of days any patient experienced delayed discharge from hospital	Q	NHSE/I
DTOC (log)	Natural logarithm of DTOC(days)	Q	NHSE/I
**Explanatory**:			
UEC Vanguards	1 if one of 29 LA's participating in this Vanguard and it is onwards and after 2015 quarter 3, 0 – otherwise	F	NHSE/I
Care home beds(log)	Natural logarithm of the number of care home beds available in the LA	A	CQC
CA Ratio	Percentage of all population that receives Carer's Allowance	Q	ONS
DLA Ratio(65)	Percentage of older population (65+ years) that receives Disability Living Allowance	Q	ONS
JSA Ratio	Percentage of all population that receives Jobseeker's Allowance	Q	ONS
PC Ratio(65)	Percentage of older population (65+ years) that receives Pension Credit	Q	ONS
Population(log)	Natural logarithm of the LA's population	A	ONS
65+ ratio	Percentage of older people (65+) in the population	A	ONS
House prices(log)	Natural logarithm of average house prices in LA	Q	LandReg
Owned house	Percentage of older people (65+) that own a house with mortgage, 2011 data	F	ONS
Owned house (outright)	Percentage of older people (65+) that own a house outright, 2011 data	F	ONS
LA type	Type of Local Authority: Metropolitan, Unitary, London and County	F	Gov
Area	Local Authority's size (m^2)	F	Gov
Rurality	Percentage of people living in rural areas and hub towns, 2011 data	F	ONS
CCGs per LA	Number of CCGs each LA is interacting with	F	Gov
CCG dummy	1 – April 2013 onwards, identifies creation of CCG's, 0 – before April 2013	F	Gov

Note: Q – quarterly, A – annual, F – fixed; NHSE/I – NHS England and Improvement, CQC – Care Quality Commission, ONS – Office for National Statistics, LandReg – HM Land Registry's Price Paid Data, Gov – UK government's online service.

There were no missing values for all data points used throughout the chosen study time period.

For peer review only

Table A2: Synthetic control estimation outcome (DTOC) measures by period:

Period	DTOC days Treated	DTOC days Control	Difference	Difference in % (to Treated)
1	1569.36	1557.42	-11.938	-0.767
2	1772.96	1761.47	-11.488	-0.652
3	1778.4	1767.08	-11.316	-0.640
4	1972.96	1961.93	-11.034	-0.562
5	1918.64	1905.17	-13.474	-0.707
6	1980.32	1965.48	-14.836	-0.755
7	2029.24	2014.04	-15.199	-0.755
8	2274.52	2258.35	-16.171	-0.716
9	2164.28	2152.22	-12.065	-0.561
10	2137.16	2125.54	-11.62	-0.547
11	2062.92	2052.17	-10.752	-0.524
12	2028.56	2017.39	-11.175	-0.554
13	1996.64	1987.86	-8.779	-0.442
14	1924.96	1917.51	-7.446	-0.388
15	1826.32	1817.59	-8.735	-0.481
16	2063	2054.71	-8.288	-0.403
17	2061.84	2051.76	-10.076	-0.491
18	1860.92	1849.25	-11.666	-0.631
19	1573.04	1561.97	-11.075	-0.709
**20**	**1701.48**	**1796.38**	**94.902**	**5.283**
**21**	**1693.04**	**2283.54**	**590.501**	**25.859**
**22**	**1873.08**	**2305.43**	**432.352**	**18.754**
**23**	**1871.88**	**2507.84**	**635.96**	**25.359**
**24**	**2084.72**	**2529.18**	**444.455**	**17.573**
**25**	**2135**	**2629.65**	**494.654**	**18.811**
**26**	**1890.96**	**2443.09**	**552.133**	**22.600**
**27**	**1819**	**2414.3**	**595.298**	**24.657**
**28**	**1878.28**	**2534.68**	**656.396**	**25.897**
**29**	**1767.72**	**2338.69**	**570.973**	**24.414**

Note: Nominal UEC Vanguards start date was 3rd quarter of 2015, quarter 20 in our dataset, results after the start of this Vanguard indicated in bold.

For peer review only

Table A3: Synthetic control estimation predictor balance:

Variable	Treated	Synthetic
JSA ratio	0.039029	0.039353
PC ratio (65+)	0.269154	0.274212
CA ratio	0.011704	0.012411
DLA ratio (65+)	0.098542	0.106904
Care home beds (log)	7.602731	7.384806
Population (log)	12.43287	12.34926
Population 65+ ratio	0.173527	0.164379
Rural or hub (%)	20.8956	15.11042
No. CCGs to LA	4.84	4.199
House prices (£, log)	12.03679	11.95333
Owning house ratio (65+)	0.077565	0.076044
Owning house outright ratio (65+)	0.071337	0.070068
Area(m^2)	79959.32	96370.46

For peer review only

BMJ Open

How local partnerships to improve urgent and emergency care have impacted delayed transfers of care from hospitals in England: a synthetic control method

Journal:	*BMJ Open*
Manuscript ID	bmjopen-2021-054568.R1
Article Type:	Original research
Date Submitted by the Author:	04-Nov-2021
Complete List of Authors:	Malisauskaite, Gintare; University of Kent, Personal Social Services Research Unit<br>Jones, Karen; University of Kent Personal Social Services Research Unit<br>Allan, Stephen; University of Kent, Personal Social Services Research Unit<br>Roland, Daniel; University of Kent, Personal Social Services Research Unit<br>Birks, Yvonne; University of York, Social Policy Research Unit<br>Baxter, Kate; University of York, Social Policy Research Unit<br>Gridley, Kate; University of York, Social Policy Research Unit
**Primary Subject Heading**:	Health policy
Secondary Subject Heading:	Health economics
Keywords:	HEALTH ECONOMICS, Health policy < HEALTH SERVICES ADMINISTRATION & MANAGEMENT, Organisation of health services < HEALTH SERVICES ADMINISTRATION & MANAGEMENT

SCHOLARONE™ Manuscripts

I, the Submitting Author has the right to grant and does grant on behalf of all authors of the Work (as defined in the below author licence), an exclusive licence and/or a non-exclusive licence for contributions from authors who are: i) UK Crown employees; ii) where BMJ has agreed a CC-BY licence shall apply, and/or iii) in accordance with the terms applicable for US Federal Government officers or employees acting as part of their official duties; on a worldwide, perpetual, irrevocable, royalty-free basis to BMJ Publishing Group Ltd ("BMJ") its licensees and where the relevant Journal is co-owned by BMJ to the co-owners of the Journal, to publish the Work in this journal and any other BMJ products and to exploit all rights, as set out in our licence.

The Submitting Author accepts and understands that any supply made under these terms is made by BMJ to the Submitting Author unless you are acting as an employee on behalf of your employer or a postgraduate student of an affiliated institution which is paying any applicable article publishing charge ("APC") for Open Access articles. Where the Submitting Author wishes to make the Work available on an Open Access basis (and intends to pay the relevant APC), the terms of reuse of such Open Access shall be governed by a Creative Commons licence – details of these licences and which Creative Commons licence will apply to this Work are set out in our licence referred to above.

Other than as permitted in any relevant BMJ Author's Self Archiving Policies, I confirm this Work has not been accepted for publication elsewhere, is not being considered for publication elsewhere and does not duplicate material already published. I confirm all authors consent to publication of this Work and authorise the granting of this licence.

For peer review only

How local partnerships to improve urgent and emergency care have impacted delayed transfers of care from hospitals in England: a synthetic control method

Gintare Malisauskaite*[1], Karen Jones[1], Stephen Allan[1], Daniel Roland[1], Yvonne Birks[2], Kate Baxter[2], Kate Gridley[2]

*Correspondence: G.Malisauskaite@kent.ac.uk

Personal Social Services Research Unit (PSSRU), University of Kent, Office CC228, Cornwallis Central, Canterbury, CT2 7NF, UK

Word count: 3,446

[1] Personal Social Services Research Unit (PSSRU), School of Social Policy, Sociology and Social Research (SSPSSR), University of Kent, UK

[2] Social Policy Research Unit (SPRU), Department of Social Policy and Social Work, University of York, UK

For peer review only

Abstract

Objectives: To assess the impact of local health and social care partnerships to improve urgent and emergency care (known as Urgent and Emergency Care (UEC) Vanguards) on delayed discharges from hospital (Delayed Transfers of Care (DTOC)). Patients should be discharged from hospital when they are medically fit. However, discharges are often delayed for non-medical reasons including access to social care. One aim of UEC Vanguards was better integration of health and social care, which could lead to fewer delays. Consequently, we assessed the relationship between UEC Vanguards and DTOC in England.

Design: Using a synthetic control estimation method 29 local authorities (LAs) that were UEC Vanguards partners were averaged into a single 'treated' unit and compared with a unit created using data from LAs that were not UEC vanguards partners to estimate the impact of UEC Vanguards on DTOC. Sensitivity analysis included fixed effects panel regressions and various placebo tests.

Setting: 150 LAs in England (excluding city of London and Isles of Scilly); 29 LAs were partners in UEC Vanguards between August 2015 and March 2018.

Primary outcome measure: Quarterly data on days of DTOC at LA level for the period 2010 to 2017.

Results: Synthetic control estimation showed a large difference in DTOC days between UEC Vanguards partner LAs compared to those that were not, with on average 23.7% lower DTOC per quarter (491 DTOC days per quarter). Fixed effect panel regressions found DTOC rates lower by 43.1% (99% confidence interval: 13.8% to 72.4%) in UEC partner LAs after the start of the Vanguards programme. We found no indication of UEC partner LAs having lower DTOC rates prior to initiation of Vanguards.

For peer review only - http://bmjopen.bmj.com/site/about/guidelines.xhtml

Conclusions: The evidence indicates a sizeable statistically significant impact of UEC Vanguards on DTOC, however, more research is required to explain the underlying reasons for this relationship.

Keywords:

Delayed Transfers of Care, Urgent and Emergency Care Vanguards, integration policy

For peer review only

Strengths and limitations of this study:

- The long span of Delayed Transfers of Care (DTOC) data used from all English Local Authorities (LA) created a comprehensive picture of how Urgent and Emergency Care (UEC) Vanguards affected DTOC rates;
- The synthetic control method allowed for a comparison between UEC and non-UEC LAs to be drawn allowing observation of how UEC partner LAs would have been likely to perform in DTOC days in the absence of UEC Vanguards;
- The data did not permit the identification and estimation of the impact of different initiatives associated to the UEC Vanguards or account for differences in breadth and reach of UEC Vanguards within the partner LAs;
- The design did not permit a clear explanation of the mechanisms by which UEC Vanguards influenced reduction in DTOC days.

Background

Delayed Transfers of Care (DTOC) is a term used to describe situations where patients are medically fit to be discharged from hospital to home or further care settings but the process is delayed,(1, 2). DTOC has attracted increased attention from policy makers alongside health and social care professionals in England,(3), due to increased rates in recent years; there is an

For peer review only - http://bmjopen.bmj.com/site/about/guidelines.xhtml

estimated £820 million annual cost for the population aged 65+ (2015-2016 estimate),(4). DTOC is also associated with decreased subsequent participation in activities of daily living, frailty, aging, high comorbidity, cognitive impairment, and dependency,(5-11).

Attempts to address the costs associated with DTOC have inspired a number of innovative policy approaches to integrating social and health care, including the Better Care Fund,(12), Integrated Care Pioneers,(13) and more recently the New Models of Care – Vanguards,(14). Vanguards set out to help improve integration of services with five different approaches,(14):

- *Acute care collaboration* – linking local hospitals to improve clinical and financial viability.
- *Urgent and emergency care* – improving coordination of services and reducing pressure on A&E departments.
- *Enhanced health in care homes* – improving and integrating health, care and rehabilitation services for older people in care homes.
- *Multi-speciality community providers* – moving specialist care into the community from hospitals.
- *Integrated primary and acute care systems* – joining up GP, hospitals, community and mental health services.

This paper examines the relationship between Urgent and Emergency Care (UEC) Vanguards and DTOC rates specifically, since discharge planning from acute care in hospitals was identified as one of the challenges by the UEC Vanguards,(15). Eight UEC Vanguards were announced to take effect in July-August 2015 with a planned end date of March 2018, however a substantial number of Vanguards managed to provide enhanced services beyond that date,(16). Models of care adopted by UEC Vanguards largely aimed to better integrate the different ways that urgent and emergency care could be accessed. To facilitate service planning and design and help patients access care via the most effective and efficient routes, UEC

For peer review only - http://bmjopen.bmj.com/site/about/guidelines.xhtml

For peer review only

Vanguards were encouraged to use a tool called a Channel Shift modelling tool,(17). The tool was aimed at facilitating further integration and cooperation between health, social and community care services, and included planning for discharge from hospital from the point of admission,(14-15). Thus, DTOC was likely to be directly affected by improved discharge planning and communication between health and social care providers. In addition, DTOC rates may be a useful proxy for certain aspects of integration, and it has been previously used as a criterion for evaluating the success of health and social care integration policy initiatives,(18).

To date, research related to DTOC is somewhat scarce. Most research considers challenges related to the discharge of older people: appropriate future support, suitable discharge destinations, and how policies are put into practice,(19-22). Waiting for post-hospital care packages accounts for a large proportion of reasons for delays,(23) and lack of social care supply is considered a major part of the explanation behind DTOC,(24, 25). However, whilst delays for which social care is responsible have increased substantially, so have delays for which the NHS is deemed responsible,(26, 27). Indeed, the NHS is continuously considered responsible for the majority of delays, with internal hospital issues with planning, documentation and transport being cited as explaining increases in DTOC,(28) alongside concerns over the integration of services more generally,(29, 30). To contribute to this literature and as part of a wider study examining the role of social care in DTOC,(31) we examined the link between UEC Vanguards and DTOC rates.

Methods

Data and setting

Data were collected for 150 English Local Authorities (LAs) for the time period between 2010 quarter 4 and 2017 quarter 4 (150 LAs, 29 quarters, 4,350 observations). City of London

For peer review only - http://bmjopen.bmj.com/site/about/guidelines.xhtml

and the Isles of Scilly were excluded due to stark differences in size in comparison to other LAs. The analysis was carried out at quarterly LA level; the time frame chosen based on the availability of DTOC data at the time of analysis. The eight UEC Vanguards took effect in August 2015 (calendar year quarter 3) and included 29 Local Authorities as partners,(14). The start date of UEC Vanguards was nominal, but without more precise information on exact timings of when the programmes took effect in different locations or when it was likely to expect any impact on outcomes, we used this nominal start date as the start of the 'treatment' in our analyses.

Statistical analysis

The primary chosen method of analysis was synthetic control estimations,(32, 33). This method creates a control unit that matches the main characteristics of the treated unit so that it has a similar outcome trajectory prior to the treatment. This then allows observation of how the 'untreated' control unit would have performed in the time following the start of the treatment, providing a comparison with the treated unit. In this case the 'treatment' was participation in UEC Vanguard programme and the 'treated unit' refers to derived averages of the outcome measure and control variables,(34) of all 29 LA partners of UEC Vanguards partner sites, i.e. the 29 LAs at each quarter were used in the sample to create a single treated unit over time. The control unit was created using the remaining LAs in England (i.e. non-UEC Vanguard partners) by estimating different weights, chosen automatically by the synthetic control estimation algorithm, for LAs to account for changes in confounding variables over time as well as across LAs. Further, we controlled for the outcome variable at each quarter prior to the treatment to achieve close tracking between the two units over the period prior to the treatment. This allowed for a credible prediction of the counterfactual i.e. what would have happened in the absence of the Vanguards programme in the UEC partner LAs. DTOC trends not being

For peer review only

For peer review only - http://bmjopen.bmj.com/site/about/guidelines.xhtml

parallel prior to Vanguards makes synthetic control estimation a preferred method for evaluation since it does not require parallel trends assumption to hold.

The outcome measure used for synthetic control estimations was the number of DTOC days (including all delayed discharge days). The analysis controlled for the following factors associated with LA-level DTOC: (i) demographics and level of need (carer's allowance, disability living allowance, total population in LA, and percentage of population above 65 years old), (ii) LA structure (type, size in square metres, percentage of people living in rural areas, the number of Clinical Commissioning Groups (CCGs) each LA is in partnership with, and care home bed supply), and (iii) economic variables (jobseeker's allowance, pension credit, house prices, percentage of single occupancy older people home ownership. A similar set of control variables were selected by Roland et al.,(35) based on findings from the literature,(5-9,19-23) concerning confounders of delayed discharges from hospitals and data availability. Further details of the data are available in Appendix Table A1.

Sensitivity analysis

As a sensitivity check, we also estimated two-way fixed effects panel regressions (Difference-in-Difference approach). Any statistically significant results would further strengthen the argument of the existence of the relationship between UEC Vanguards and DTOC. This provided an average estimate of the effect size of the Vanguards programme on DTOC . The model specification used was:

$$DTOC_{it} = \alpha_i + V_{it}\beta_1 + V_{it}T_t\beta_2 + C_{it}\beta_3 + u_{it} \quad (1)$$

Where the dependent variable $DTOC_{it}$ is expressed as the natural logarithm of the number of DTOC days, to mitigate the potential effect of large outliers and data skewness in regression results. Subscript i indicates a Local Authority, t time (in quarters) and α is a LA dependent intercept. Vanguard partners were identified using the dummy variable V_{it} (1 = after programme start, 2015 quarter 3, 0 = before or not Vanguard partner), and β_1 is the coefficient

For peer review only - http://bmjopen.bmj.com/site/about/guidelines.xhtml

of interest for the analysis, showing the average effect of being a partner in the UEC Vanguards on DTOC. We also included interactions between participation in UEC Vanguards and time quarters $V_{it}T_t\beta_2$, with β_2 being a vector of coefficients associated with them. Finally, C_{it} is a vector of the same control variables used in the synthetic control estimation and quarter dummies for each quarter in the sample, with β_3 being a vector of coefficients associated with them, representing the effects on DTOC rate.

The equation (1) was estimated using fixed effects panel regressions,(36). A Hausman test found that a fixed effects model was preferred over a random effects model (Hausman test statistic of 46.30, significant at 1%). Some variables were dropped during the estimation process due to the invariant nature of some data (e.g. LA type and size etc.). All regressions used cluster-robust standard errors centred on LAs, to account for potential heteroscedasticity and unobservable characteristics which could make LA level clusters more similar.

Finally, given the potential for selection bias into UEC vanguards, as robustness checks we also conducted placebo tests to check if there was any evidence of significant differences in DTOC rates between UEC and non-UEC LAs by a) assuming that the UEC programme was in existence across the whole period of analysis and b) using data from prior to the start of the programme. These robustness checks were estimated using Ordinary Least Squares (OLS) and random effects (RE) given UEC vanguard partners were assumed fixed over time.

Patient and Public Involvement statement

Both public and practice stakeholders were involved in the project as part of the steering committee. They helped to shape initial study design and participated in the discussion of initial and final findings alongside dissemination. Further stakeholder engagement through dissemination at practice facing events looking at findings further shaped the presentation of early findings.

For peer review only

Results

[Table 1]

[Table 2]

Descriptive statistics

Table 1 presents descriptive statistics from the last quarter of 2010 to the last quarter of 2017. The average number of DTOC days was 2,755 per English LA per quarter. 6.7% of the sample observations were UEC Vanguard partner sites. Table 2 provides more detail on outcome and control variables based on participation in UEC Vanguard programme presenting mean values before and after the start of UEC Vanguards. Before and after means for control variables appear similar for both UEC and non-UEC partner sites, the main noteworthy difference being the sizeable increase in DTOC days after the start of the Vanguards in non-UEC LAs.

[Figure 1]

Figure 1 compares average number of DTOC days (ratio to LA population) per quarter for UEC partner LAs compared to those non-UEC LAs. The increase in DTOC days after the start of the UEC programme, obvious for the non-UEC LAs, was hardly visible for LAs in UEC Vanguards. However, since the trends prior to the Vanguards seem non-parallel, robustness checks were employed to check for any indication that selection into the UEC Vanguards programme was based on DTOC rates.

Main results

Figure 2 depicts the predictions of synthetic control estimations. The figure shows that from the beginning of the UEC Vanguards programme, partner sites had consistently lower than average DTOC rates than would have been predicted without the Vanguards. This difference was sizeable, with on average 491 fewer DTOC days per quarter per LA, or around 23.7% less (estimation outcome matrix, showing actual average days of DTOC for UEC partner LAs and

For peer review only

For peer review only - http://bmjopen.bmj.com/site/about/guidelines.xhtml

predicted synthetic control unit days of DTOC, is provided in Table A2 in the Appendix). The synthetic control tracking the data prior to UEC programme indicates a good fit for the model (the predictor balances between averaged treated unit and synthetic control unit are available in Table A3 in the Appendix) and standardized p-values indicate the quarterly effects are significant at 1% level (synthetic control post-estimation results in Table A4 in the Appendix),(32, 37). The sizeable dip in DTOC days just prior to the start of UEC Vanguards of, on average, 300 delayed days less per UEC Vanguard participating LA compared to the previous quarter, suggests that some form of preparation with regard to integration and DTOC could have taken place in the participating sites.

[Figure 2]

Sensitivity analysis

The first column of Table 3 reports the full results from the fixed effects panel regression estimation and shows UEC Vanguards are associated with 43.1% lower DTOC rates at 1% significance level (99% confidence interval: 13.8-72.4%), and there was no indication of any trend differences between UEC partner and non-partner LAs after the start of the programme. It was a preferred estimation for finding the coefficient size associated with UEC Vanguards impact, since when controlling for LA level characteristics Hausman test revealed systematic differences between fixed effects and random effects models and fixed effects model has an advantage of controlling for any other unobservable LA level characteristics that are fixed in time.

[Table 3]

One of the possible interpretations for the observed results may be that sites participating in UEC Vanguards could have been selected based on better DTOC rates. There was no publicly available information on specific selection criteria but testing for statistical differences between DTOC rates was carried out by running the model using two modifications:

For peer review only - http://bmjopen.bmj.com/site/about/guidelines.xhtml

a) without identifying the start of the UEC Vanguard, checking for an overall difference between UEC participating sites and others over the entire period of time of data;

b) using only data prior to 2015, the start of the initiative.

The findings (Table 3, columns a) and b) respectively) showed there was no statistically significant relationship between UEC Vanguards and DTOC in these specifications. Overall marginal effects of UEC Vanguards for these specifications were also consistently statistically insignificant. This suggests that the difference in DTOC rates between UEC and non-UEC partner LAs only becomes significant after the start of UEC programme.

Discussion

Main findings

Our findings suggest that LAs that were part of UEC Vanguards had significantly lower average DTOC than non-UEC sites after the start of the programme. Overall DTOC rates rose substantially in the second quarter of 2015 until the end of 2016, potentially explained by severe cuts to the funding of social care,(38); however, they rose significantly less, if at all, in UEC Vanguard sites. We found no evidence suggesting that UEC Vanguard sites had lower DTOC rates prior to becoming Vanguards but are unable to rule out the possibility that they might have been in a better position to reduce DTOC due to other as yet unidentified factors, e.g. other health care programmes already in place in the UEC partner LAs. It is difficult to pinpoint the reasons why UEC Vanguards influenced DTOC rates. One potential explanation from policies and initiatives associated with being in a Vanguard is the Channel Shift modelling tool-kit, which supports integration of services, communication and cooperation between hospital and community based services,(18). Channel Shift interventions include planning discharge from time of admission, discharging for further assessment ('discharge to assess')

For peer review only

and rapid response services, yet we could not formally test if this indeed is the main reason for the effect found.

There is limited previous research that has looked at the relationship between New Models of Care – Vanguards and DTOC rates. One study reported that Vanguards are associated with a small reduction in hospital admissions,(39). This highlights important implications for the National Health Service and social care, alongside individuals discharged from hospital. The move towards greater integration of services is not a new idea, and the importance of local collaboration has been consistently stressed,(40). However, it is difficult to isolate the impact of the UEC Vanguards from other Government integration policies, together with the austerity climate facing the care sector. This is particularly important with regard to potential changes the COVID-19 response has led to, including hospital discharge service, which is expected to influence the further development of discharge to assess and integration of health and social care services,(41). Although New Care Models were discontinued well before the start of the global pandemic, assumptions were made that practices adopted during the time of New Care Models activity period would continue to be used,(16). This makes it reasonable to expect this programme could have made a positive contribution towards freeing hospital beds for Covid-19 patients, however further research would be necessary to establish the size and significance of any impact. Alongside our findings, this indicates that the Vanguards programme should be of interest to policy makers in terms of lessons learned for dealing with discharges during and following the COVID-19 pandemic.

Limitations

The synthetic control estimation suggests an element of causality between UEC participation and DTOC rates. However, a number of limitations need to be acknowledged. Firstly, we could not account for selection criteria into the Vanguards, different initiatives within UEC Vanguards and the impact of other active health policies potentially influencing

For peer review only - http://bmjopen.bmj.com/site/about/guidelines.xhtml

the results during the Vanguards' activity period. Such information could further inform analysis and provide further context for this finding. Further work on specific policies used within UEC Vanguard sites, including qualitative analysis and more detailed examination of specific UEC Vanguards' mechanisms of action could help untangle potential reasons for the association between UEC Vanguards and DTOC.

Secondly, this analysis does not account for different timeframes in which UEC Vanguards took effect in different locations, differences within the eight UEC Vanguards, or possible different levels of exposure to UEC Vanguard influence in each LA. We anticipate there was some variation in associations between different UEC Vanguards and outcomes. However, this approach should be sufficient to show the average effect of the UEC Vanguards programme.

Thirdly, we did not look into re-admission rates for UEC Vanguard partner sites. Evidence suggests that there is an association between high bed occupancy and re-admission rates into hospitals in England, likely due to increased rates of discharge when bed occupancy is high,(42). There remains the possibility that hospitals in UEC partner LAs may have had higher readmission rates because of higher bed occupancy and/or improved rates of discharge. If so, the positive benefit of the UEC Vanguard programme would be mitigated somewhat. However, the UEC Vanguard programme directly addressed one of the likely causes of readmissions from high bed occupancy, i.e. inadequate discharge planning. The identified limitations could be further routes of enquiry, even if achieving precision in quantifying health care programmes is unlikely.

A potential solution to refining the analysis would be to use individual level data to conduct a similar analysis. This could allow more precise estimation of the effect size as case-specific confounders could be taken into account, which is not possible at an aggregated level.

For peer review only - http://bmjopen.bmj.com/site/about/guidelines.xhtml

Overall, the estimation methods adopted revealed a strong association between the UEC Vanguard and a reduction of DTOC, which advocates the success of this integration programme and should encourage further research to reveal which specific aspects of this initiative were particularly beneficial, which could guide further policy decisions.

References

1. NHS England. Delayed Transfers of Care. Leeds: NHS England; 2011 [updated 2019]. https://www.england.nhs.uk/statistics/statistical-work-areas/delayed-transfers-of-care/. Accessed Oct 2019.
2. The Kings Fund. Delayed transfers of care: a quick guide. London: The Kings Fund; 2018. https://www.kingsfund.org.uk/publications/delayed-transfers-care-quick-guide. Accessed Nov 2020.
3. NHS Providers. The rise of delayed transfers of care. London: NHS Providers; 2017. https://nhsproviders.org/the-rise-of-delayed-transfers-of-care. Accessed Oct 2019.
4. National Audit Office. Discharging older patients from hospitals. London: National Audit Office; 2016. https://www.nao.org.uk/wp-content/uploads/2015/12/Discharging-older-patients-from-hospital.pdf. Accessed Apr 2019.
5. Bo M, Fonte G, Pivaro F, et al. Prevalence of and factors associated with prolonged length of stay in older hospitalized medical patients. *GeriatrGerontolInt*2016;16(3):314-321.
6. Challis D, Hughes J, Xie C, et al. An examination of factors influencing delayed discharge of older people from hospital. *IntJGeriatrPsychiatry*2014;29(2):160-168.
7. Costa AP, Hirdes JP. Clinical characteristics and service needs of alternate-level-of-care patients waiting for long-term care in Ontario hospitals. *HealthcPolicy* 2010;6(1):32-46.
8. Costa AP, Poss JW, Pierce T, et al. Acute care inpatients with long-term delayed-discharge: Evidence from a Canadian health region. *BMCHealthServRes*2012;12:172-182.
9. McClaran J, Tover R, Duarte E. Long hospital stays and need for alternate level of care at discharge. Does family make a difference for elderly patients? *CanFamPhysician*1996;42:449-461.
10. Rojas-García A, Turner S, Pizzo E, et al. Impact and experiences of delayed discharge: A mixed-studies systematic review. *HealthExpect*2018;21(1):41-56.
11. Bai AD, Dai C, Srivastava S, et al. Risk factors, costs and complications of delayed hospital discharge from internal medicine wards at a Canadian academic medical centre: retrospective cohort study. *BMCHealthServRes*2019; doi: 10.1186/s12913-019-4760-3.
12. NHS England. Better Care Fund. London: NHS England; 2013. https://www.england.nhs.uk/ourwork/part-rel/transformation-fund/bcf-plan/. Accessed Oct 2019.

For peer review only - http://bmjopen.bmj.com/site/about/guidelines.xhtml

13. NHS England. Integrated Care Pioneers. London: NHS England; 2014. https://www.england.nhs.uk/new-care-models/integrated-care-pioneers/. Accessed Oct 2019.

14. NHS England. New Care Models: Vanguards – developing blueprint for the future of NHS and care services. London: NHS England; 2016. https://www.england.nhs.uk/wp-content/uploads/2015/11/new_care_models.pdf. Accessed Mar 2019.

15. NHS England. New Care Models: supporting the vanguards Urgent and Emergency Care (UEC) vanguards: summary. London: NHS England; 2015. https://www.england.nhs.uk/wp-content/uploads/2015/12/uec-support-package-summary.pdf. Accessed Oct 2019.

16. National Health Executive. Vanguards: Stevens pledges 10-year plan, original aims failed due to trust deficits. 2018. https://www.nationalhealthexecutive.com/Health-Care-News/vanguards-stevens-pledges-10-year-plan-original-aims-failed-due-to-trust-deficits

17. NHS England. Urgent and emergency care channel shift model. London: NHS England; 2015. https://www.england.nhs.uk/urgent-emergency-care/about-uec/uec-channel-shift/. Accessed Oct 2019.

18. Forder J, Caiels J, Harlock J, et al. A system-level evaluation of the Better Care Fund: Final report. Personal Social Services Research Unit; 2018. https://www.pssru.ac.uk/pub/5424.pdf. Accessed Oct 2019.

19. Coffey A. Discharging older people from hospital to home: what do we know? Int. *JOlderPeopleNurs*2006;1:141-150.

20. Glasby J, Littlechild R, Pryce K. All dressed up and nowhere to go? Delayed hospital discharges and older people. *JHealthServResPol*2006;11(1):52-58.

21. Swinkels A, Mitchell T. Delayed transfer from hospital to community settings: the older person's perspective. *HealthSocCareCommunity*2008;17(1):45-53.

22. Shaw J, Kontos P, Martin W, et al. The inter-relation between policy and practice for transitions from hospital to home: an ethnographic case study in England's National Health Service. *BMCHealthServRes*2014;P111. doi:10.1186/1472-6963-14-S2-P111.

23. The Nuffield Trust. Delayed transfers of care. We look at delayed transfers of care for patients following NHS treatment. 2021. The Nuffield Trust. https://www.nuffieldtrust.org.uk/resource/delayed-transfers-of-care. Accessed May 2021.

24. Gaughan J, Gravelle H, Siciliani L. Testing the bed-blocking hypothesis: does nursing and care home supply reduce delayed hospital discharges? J*HealthEcon*2015;24(1):32-44.

25. Allan S, Roland D, Malisauskaite G, et al. The influence of home care supply on delayed discharges from hospital in England. *BMCHealthServRes*2021:[forthcoming].

26. Maguire D. Delayed transfers of care: a target that misses the mark? 2018. The King's Fund. Blog. https://www.kingsfund.org.uk/blog/2018/01/delayed-transfers-care-target-misses-mark. Accessed May 2021.

27. Bottery S. Stopping the blame game around delayed transfers of care. 2017. King's Fund. Blog. https://www.kingsfund.org.uk/blog/2017/11/stopping-blame-game-delayed-transfers-care. Accessed May 2021.

For peer review only - http://bmjopen.bmj.com/site/about/guidelines.xhtml

28. Edwards N. What's behind delayed transfers of care? 2017. The Nuffield Trust. Briefing. https://www.nuffieldtrust.org.uk/resource/what-s-behind-delayed-transfers-of-care. Accessed May 2021.

29. Fernandez J, McGuire A, Raikou M. Hospital coordination and integration with social care in England: The effect on post-operative length of stay. *JHealthEcon*2018;61:233-243.

30. Goddard M, Mason A. Integrated care: A pill for all ills? *Int. JHealthPolicyManag*2017;6(1):1-3.

31. Jones K, Allan S, Malisauskaite G, et al. The influence of social care on delayed transfers of care (DTOC) among older people: Final report. Personal Social Services Research Unit. 2020. https://kar.kent.ac.uk/id/eprint/77826. [In press].

32. Abadie A, Diamond A, Hainmueller J. Synthetic Control Methods for Comparative Case Studies: Estimating the Effect of California's Tobacco Control Program. *JAmStatAssoc*2010;105(490):493-505.

33. Abadie A, Diamond A, Hainmueller J. Comparative Politics and the Synthetic Control Method. *AmJPoliticalSci*2015;59(2):495-510.

34. Kreif N, Grieve R, Hangartner D, et al. Examination of the Synthetic Control Method for Evaluating Health Policies with Multiple Treated Units. *HealthEcon*2015;25(12):1514-1528.

35. Roland D, Malisauskaite G, Jones K, et al. Identifying unexplained DTOC at Local Authority level. 2020. [Forthcoming].

36. Wooldridge J M. Econometric analysis of cross section and panel data. 2nd ed. Cambridge: The MIT Press; 2010.

37. Galiani S, Quistorff B, (2017), The synth runner package: Utilities to automate synthetic control estimation using synth. *SJ*2017:17(4):834-849.

38. Bate A. Delayed transfers of care in the NHS. Briefing paper Nr. 7415, London: House of Commons Library; 2017. https://commonslibrary.parliament.uk/research-briefings/cbp-7415/. Accessed Oct 2019.

39. Morciano M, Checkland K, Billings J, et al. New integrated care models in England associated with small reduction in hospital admissions in longer-term: a difference-in-differences analysis. *HealthPolicy*2020;124(8):826-833.

40. NHS England. Why not home? Why not today? Behind every delayed transfer of care from hospital to home, there is a person, in the wrong place at the wrong time. Monthly Delayed Transfers of Care Situation Report. London: NHS England; 2018. https://www.england.nhs.uk/statistics/wp-content/uploads/sites/2/2018/11/Monthly-Delayed-Transfers-of-Care-Situation-Report-Principles-Definitions-and-Guidance.pdf. Accessed Jun 2020.

41. HM Government. Hospital Discharge Service: Policy and Operating Model. London: Department of Health and Social Care; 2020. https://assets.publishing.service.gov.uk/government/uploads/system/uploads/attachment_data/file/912199/Hospital_Discharge_Policy_1.pdf. Accessed Nov 2020.

42. Friebel R, Fisher R, Deeny SR, et al. The implications of high bed occupancy rates on readmission rates in England: A longitudinal study. *HealthPolicy*2019:123(8):765-772.

For peer review only - http://bmjopen.bmj.com/site/about/guidelines.xhtml

Abbreviations

UEC: Urgent and emergency care vanguard;

DTOC: delayed transfers of care;

LA: Local Authority;

CCG: Clinical Commissioning Group;

FE: Fixed effects panel regression model.

OLS: Ordinary least squares;

Ethics and dissemination

Ethical approval was not required for this study due to use of publicly available secondary data. Work discussed in this article has been presented in research seminars, conferences and advisory committee meetings.

Author Contributions

GM conceived the statistical methodology, performed the statistical analysis and drafted the manuscript; KJ contributed to the conception, study design and the final writing of this paper; SA contributed to the study design, statistical methodology and the final writing of this paper; DR contributed to the statistical methodology and the final writing of this paper; YB critically revised the draft and contributed to the final writing of this paper; KB critically revised the draft and contributed to the final writing of this paper; KG critically revised the draft and contributed to the final writing of this paper.

Funding

For peer review only - http://bmjopen.bmj.com/site/about/guidelines.xhtml

This study was funded by the National Institute for Health Research School for Social Care Research, grant number C088/CM/UKJB-P116. The views expressed in this independent research publication are those of the authors and not necessarily those of the NIHR SSCR, the National Institute for Health Research or the Department of Health and Social Care.

Competing interests

The authors declare that they have no competing interests.

Data statement

Technical appendix, statistical code, and dataset available from the corresponding author on reasonable request.

[Appendix]

For peer review only

For peer review only - http://bmjopen.bmj.com/site/about/guidelines.xhtml

Tables and Figures

Table 1: Descriptive statistics

Variable	Mean	SD	Min/Max
**Dependent variables:**			
DTOC days	2755.394	3124.008	0/26733
DTOC(log)	7.438	0.986	0/10.194
**Explanatory variables:**			
UEC Vanguard	0.067	0.249	0/1
JSA ratio	0.026	0.016	0.001/0.09
PC ratio (65+)	0.235	0.096	0.062/0.691
CA ratio	0.011	0.004	0.004/0.027
DLA ratio (65+)	0.087	0.040	0.025/0.257
Care home beds (log)	7.553	0.825	5.451/9.461
Population (log)	12.598	0.606	10.519/14.257
Population 65+ ratio	0.166	0.043	0.06/0.286
Rurality (%)	17.507	24.468	0/100
No. CCGs to LA	5.660	3.480	1/21
House prices (£, log)	12.343	0.519	11.443/14.62
Owning single home r. (65+)	0.075	0.022	0.013/0.126
Owning single home outright r. (65+)	0.069	0.021	0.011/0.115
Area(m^2)	86839.430	150126.3	1213/803761
CCG dummy	0.655	0.475	0/1
LA type:			
Metropolitan	0.240	0.427	0/1
London	0.213	0.410	0/1
County	0.180	0.384	0/1
No of obs.		4,350	

Note: Further information on data sources and derivation of variables available in Appendix TableA1.

For peer review only - http://bmjopen.bmj.com/site/about/guidelines.xhtml

Table 2: Means of outcome and control variables by UEC Vanguard participation, before and after the start of the programme

	Non-UEC		UEC	
	Before	After	Before	After
DTOC days	2510.955	3690.847	2130.523	2233.072
DTOC(log)	7.344334	7.758407	7.255489	7.297711
JSA ratio	0.031272	0.012509	0.038485	0.016309
PC ratio (65+)	0.254087	0.190102	0.263469	0.19152
CA ratio	0.010333	0.012434	0.011437	0.014433
DLA ratio (65+)	0.087383	0.080628	0.097996	0.090038
Care home beds (log)	7.521928	7.535296	7.666286	7.666745
Population (log)	12.60945	12.63799	12.5038	12.52737
Population 65+ ratio	0.161205	0.168712	0.174282	0.183642
House prices (£, log)	12.34778	12.52908	12.03327	12.17675
Rurality (%)	16.31793		22.46746	
No. CCGs to LA	5.752066		5.275862	
Owning single home r. (65+)	0.074886		0.077415	
Owning single home outright r. (65+)	0.068836		0.071335	
Area(m^2)	83452.44		100971.3	
No of obs.	2,420	1,210	551	290

Note: Before – 2010 q4 to 2015 q2, 19 quarters; After – 2015 q3 to 2017 q4, 10 quarters.

Further information on data sources and derivation of variables available in Appendix TableA1.

For peer review only

Table 3: Regression results with DTOC (log) as dependent variable

Variable	FE	RE		OLS	
		a)	b)	a)	b)
UEC Vanguard	-0.431***	-0.0852	-0.054	-0.101	-0.053
	[0.112]	[0.150]	[0.149]	[0.149]	[0.147]
JSA ratio	-2.949	-4.046	-0.393	1.229	10.734*
	[5.746]	[4.572]	[4.330]	[4.802]	[6.065]
PC ratio (65+)	3.559	2.783**	1.478	1.229	0.832
	[2.429]	[1.414]	[1.391]	[0.848]	[0.979]
CA ratio	31.956	-4.278	-4.517	-41.223**	-60.315**
	[51.607]	[25.682]	[29.333]	[18.047]	[26.653]
DLA ratio (65+)	-1.668	-4.941**	-2.106	-0.063	1.209
	[7.182]	[2.244]	[2.498]	[1.616]	[1.756]
Care home beds (log)	-0.539	-0.401*	-0.289	-0.187	-0.264
	[0.353]	[0.213]	[0.229]	[0.161]	[0.197]
Population (log)	1.950	1.658***	1.629***	1.405***	1.540***
	[2.111]	[0.266]	[0.269]	[0.198]	[0.239]
Population 65+ ratio	16.315	12.130**	11.702**	2.336	2.641
	[10.441]	[5.602]	[5.950]	[3.246]	[4.652]
House prices (£, log)	0.028	0.107	0.161	0.070	0.195
	[0.263]	[0.146]	[0.136]	[0.133]	[0.159]
CCG dummy	0.317	0.363*	-0.034	0.715**	0.556***
	[0.419]	[0.216]	[0.123]	[0.159]	[0.149]
Rurality (%)	-	-0.009	-0.010	-0.005	-0.006
		[0.006]	[0.008]	[0.005]	[0.008]
No. CCGs to LA	-	-0.024*	-0.033**	-0.031***	-0.033**
		[0.014]	[0.015]	[0.012]	[0.014]
Home single ownership r. (65+)	-	-105.012*	-160.919***	-74.033*	-126.771**
		[56.140]	[61.456]	[40.551]	[50.798]
Home single ownership outright r. (65+)	-	104.434*	160.952***	81.398*	137.968***
		[56.140]	[60.303]	[41.406]	[50.871]
Area(m^2)	-	-3.25e-08	7.95e-09	1.86e-07	2.30e-07
		[4.76e-07]	[7.34e-07]	[5.48e-07]	[8.33e-07]
LA Type:					
Metropolitan	-	-0.308**	-0.431***	-0.202*	-0.345***
		[0.152]	[0.140]	[0.103]	[0.123]
London	-	-0.502***	-0.586***	-0.602***	-0.665***
		[0.159]	[0.162]	[0.120]	[0.142]
County	-	0.149	0.120	0.230	0.211
		[0.203]	[0.211]	[0.183]	[0.203]
Cons.	-17.234	-10.257***	-13.516***	-10.062***	-12.676***
	[28.685]	[2.839]	[2.592]	[2.181]	[2.629]
Interactions between time quarters and UEC Vanguard	Yes	Yes	Yes	Yes	Yes
Time quarter dummies	Yes	Yes	Yes	Yes	Yes
No. of obs.	4,350	4,350	2550	4,350	2550
No. of groups	150	150		150	

Note: Robust clustered standard errors in square brackets underneath the coefficients. Variables omitted during the FE estimation process due to fixed effects nature of the model: LAs' rurality (%), no. CCGs to LA, owning single home ratio (65+), owning single home outright ratio (65+), area(m^2), LA type; more details of variable construction available in the Appendix; model also includes year indicators between 2010-2017.

Robustness checks in RE and OLS columns: a) UEC Vanguard partner LAs are identified as being active with a binary variable across the whole period of analysis; b) UEC Vanguard partner LAs are identified with a binary variable only using sample prior to 2015. FE results not available for these specifications due to treatment being fixed across time in the estimations.

*** - p<1%, ** - p<5%, * - p<10%

For peer review only - http://bmjopen.bmj.com/site/about/guidelines.xhtml

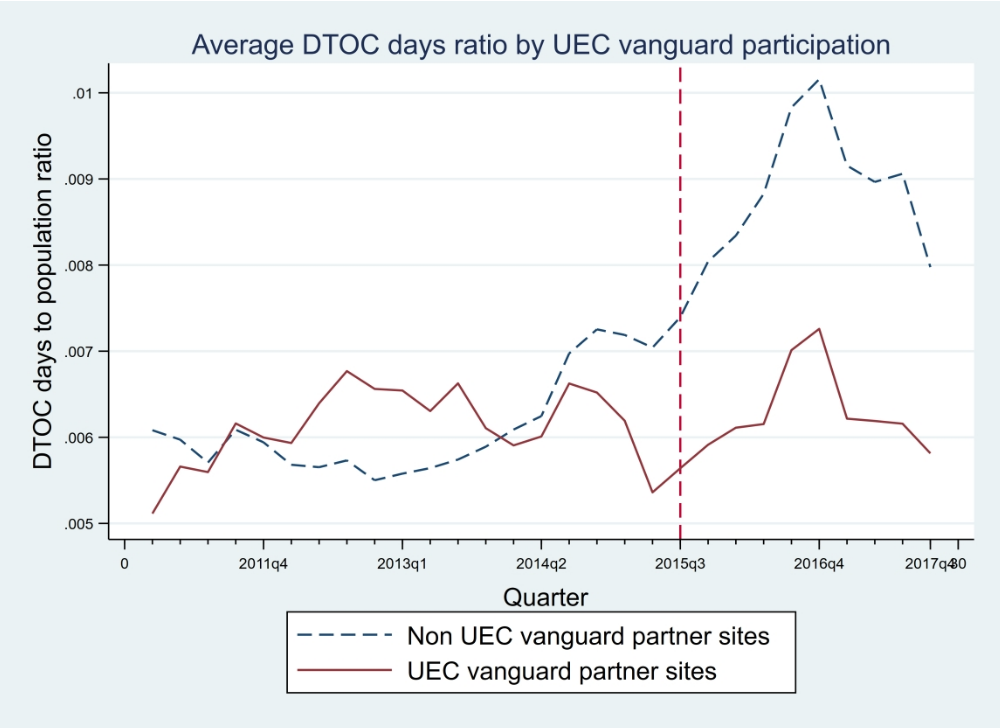

Figure 1: Average DTOC days over time

330x240mm (300 x 300 DPI)

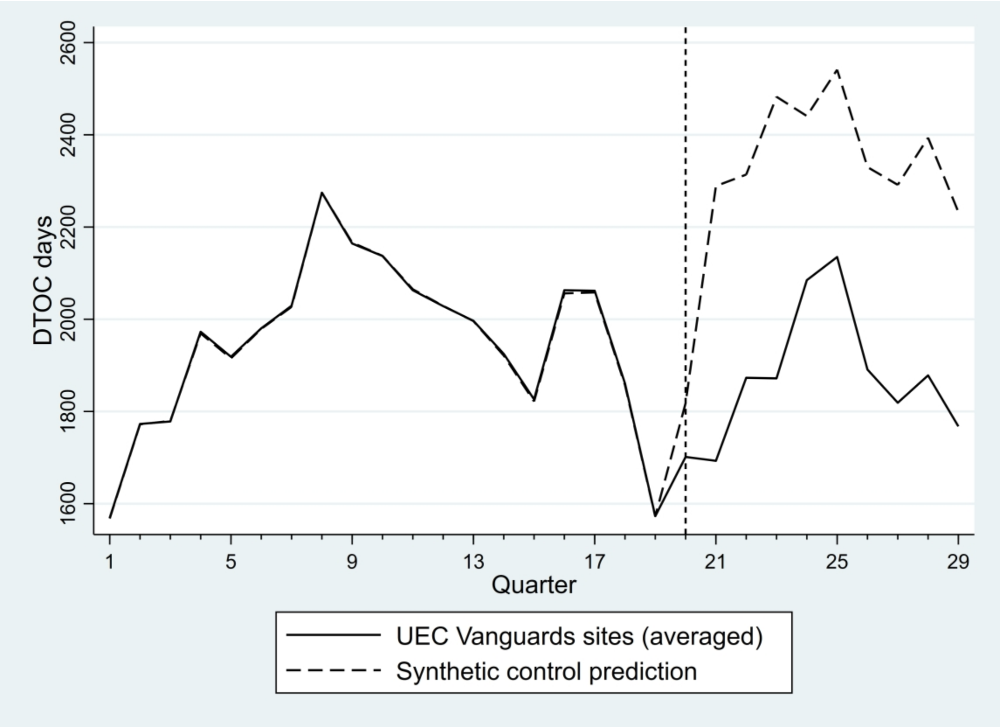

Figure 2: Synthetic control estimations

330x240mm (300 x 300 DPI)

Appendix

Table A1: Definition of variables

Variable	Explanation	Frequency	Source
**Dependent**:			
DTOC (days)	Number of days any patient experienced delayed discharge from hospital	Q	NHSE/I
DTOC (log)	Natural logarithm of DTOC(days)	Q	NHSE/I
**Explanatory**:			
UEC Vanguards	1 if one of 29 LA's participating in this Vanguard and it is onwards and after 2015 quarter 3, 0 – otherwise	F	NHSE/I
Care home beds(log)	Natural logarithm of the number of care home beds available in the LA	A	CQC
CA Ratio	Percentage of all population that receives Carer's Allowance	Q	ONS
DLA Ratio(65)	Percentage of older population (65+ years) that receives Disability Living Allowance	Q	ONS
JSA Ratio	Percentage of all population that receives Jobseeker's Allowance	Q	ONS
PC Ratio(65)	Percentage of older population (65+ years) that receives Pension Credit	Q	ONS
Population(log)	Natural logarithm of the LA's population	A	ONS
65+ ratio	Percentage of older people (65+) in the population	A	ONS
House prices(log)	Natural logarithm of average house prices in LA	Q	LandReg
Owned house	Percentage of single occupancy households of older people (65+) that own a house with mortgage, 2011 data	F	ONS
Owned house (outright)	Percentage of single occupancy households of older people (65+) that own a house outright, 2011 data	F	ONS
LA type	Type of Local Authority: Metropolitan, Unitary, London and County	F	Gov
Area	Local Authority's size (m^2)	F	Gov
Rurality	Percentage of people living in rural areas and hub towns, 2011 data	F	ONS
CCGs per LA	Number of CCGs each LA is interacting with	F	Gov
CCG dummy	1 – April 2013 onwards, identifies creation of CCG's, 0 – before April 2013	F	Gov

Note: Q – quarterly, A – annual, F – fixed; NHSE/I – NHS England and Improvement, CQC – Care Quality Commission, ONS – Office for National Statistics, LandReg – HM Land Registry's Price Paid Data, Gov – UK government's online service.

There were no missing values for all data points used throughout the chosen study time period.

For peer review only

Table A2: Synthetic control estimation outcome (DTOC) measures by period:

Period	DTOC days Treated	DTOC days Control (synth)	Difference	Difference in % (to Treated)
1	1569.36	1567.916	-1.444	-0.09201
2	1772.96	1772.207	-0.753	-0.04247
3	1778.4	1779.567	1.167	0.065621
4	1972.96	1969.42	-3.54	-0.17943
5	1918.64	1915.392	-3.248	-0.16929
6	1980.32	1979.21	-1.11	-0.05605
7	2029.24	2026.926	-2.314	-0.11403
8	2274.52	2274.597	0.077	0.003385
9	2164.28	2166.296	2.016	0.093149
10	2137.16	2137.553	0.393	0.018389
11	2062.92	2065.035	2.115	0.102525
12	2028.56	2029.041	0.481	0.023711
13	1996.64	1996.134	-0.506	-0.02534
14	1924.96	1921.385	-3.575	-0.18572
15	1826.32	1821.821	-4.499	-0.24634
16	2063	2056.206	-6.794	-0.32933
17	2061.84	2058.22	-3.62	-0.17557
18	1860.92	1857.615	-3.305	-0.1776
19	1573.04	1572.29	-0.75	-0.04768
**20**	**1701.48**	**1818.133**	**116.653**	**6.855972**
**21**	**1693.04**	**2288.897**	**595.857**	**35.1945**
**22**	**1873.08**	**2313.684**	**440.604**	**23.52297**
**23**	**1871.88**	**2482.475**	**610.595**	**32.61935**
**24**	**2084.72**	**2440.76**	**356.04**	**17.07855**
**25**	**2135**	**2541.185**	**406.185**	**19.02506**
**26**	**1890.96**	**2330.016**	**439.056**	**23.21868**
**27**	**1819**	**2291.485**	**472.485**	**25.97499**
**28**	**1878.28**	**2393.571**	**515.291**	**27.4342**
**29**	**1767.72**	**2232.34**	**464.62**	**26.28357**

Note: Nominal UEC Vanguards start date was 3rd quarter of 2015, quarter 20 in our dataset, results after the start of this Vanguard indicated in bold.

For peer review only

Table A3: Synthetic control estimation predictor balance:

Variable	Treated	Synthetic
JSA ratio	0.039029	0.040455
PC ratio (65+)	0.269154	0.28076
CA ratio	0.011704	0.012353
DLA ratio (65+)	0.098542	0.103666
Care home beds (log)	7.602731	7.35620
Population (log)	12.43287	12.35283
Population 65+ ratio	0.173527	0.160181
Rural or hub (%)	20.8956	12.78465
No. CCGs to LA	4.84	3.924
House prices (£, log)	12.03679	11.99105
Owning single home r. (65+)	0.077565	0.07411
Owning single home outright r. (65+)	0.071337	0.068227
Area(m^2)	79959.32	76051.94

Table A4: Synthetic control post-estimation results:

Post estimation period	Estimates (synth-treated)	p-vals	p-vals standardized
c1 (quarter 20)	-116.653	0.77686	0
c2 (quarter 21)	-595.857	0.38843	0
c3 (quarter 22)	-440.604	0.545455	0
c4 (quarter 23)	-610.595	0.446281	0
c5 (quarter 24)	-356.04	0.719008	0
c6 (quarter 25)	-406.185	0.710744	0
c7 (quarter 26)	-439.056	0.677686	0
c8 (quarter 27)	-472.485	0.628099	0
c9 (quarter 28)	-515.291	0.603306	0
c10 (quarter 29)	-464.62	0.603306	0

Note: post-estimation results using synth_runner command on Stata.

For peer review only

STROBE Statement—Checklist of items that should be included in reports of ***cross-sectional studies***

	Item No	Recommendation	Page No
**Title and abstract**	1	(*a*) Indicate the study's design with a commonly used term in the title or the abstract	1
		(*b*) Provide in the abstract an informative and balanced summary of what was done and what was found	2
**Introduction**			
Background/rationale	2	Explain the scientific background and rationale for the investigation being reported	4-5
Objectives	3	State specific objectives, including any prespecified hypotheses	5
**Methods**			
Study design	4	Present key elements of study design early in the paper	2, 6
Setting	5	Describe the setting, locations, and relevant dates, including periods of recruitment, exposure, follow-up, and data collection	5-6
Participants	6	(*a*) Give the eligibility criteria, and the sources and methods of selection of participants	N/A
Variables	7	Clearly define all outcomes, exposures, predictors, potential confounders, and effect modifiers. Give diagnostic criteria, if applicable	6-8
Data sources/ measurement	8*	For each variable of interest, give sources of data and details of methods of assessment (measurement). Describe comparability of assessment methods if there is more than one group	Appendix A1
Bias	9	Describe any efforts to address potential sources of bias	7-8, 12-13
Study size	10	Explain how the study size was arrived at	5-6
Quantitative variables	11	Explain how quantitative variables were handled in the analyses. If applicable, describe which groupings were chosen and why	7-8
Statistical methods	12	(*a*) Describe all statistical methods, including those used to control for confounding	6-8
		(*b*) Describe any methods used to examine subgroups and interactions	N/A
		(*c*) Explain how missing data were addressed	N/A
		(*d*) If applicable, describe analytical methods taking account of sampling strategy	6
		(*e*) Describe any sensitivity analyses	7-8
**Results**			
Participants	13*	(a) Report numbers of individuals at each stage of study—eg numbers potentially eligible, examined for eligibility, confirmed eligible, included in the study, completing follow-up, and analysed	N/A
		(b) Give reasons for non-participation at each stage	N/A
		(c) Consider use of a flow diagram	N/A
Descriptive data	14*	(a) Give characteristics of study participants (eg demographic, clinical, social) and information on exposures and potential confounders	9
		(b) Indicate number of participants with missing data for each variable of interest	N/A
Outcome data	15*	Report numbers of outcome events or summary measures	9

For peer review only - http://bmjopen.bmj.com/site/about/guidelines.xhtml

Main results	16	(*a*) Give unadjusted estimates and, if applicable, confounder-adjusted estimates and their precision (eg, 95% confidence interval). Make clear which confounders were adjusted for and why they were included	9-10
		(*b*) Report category boundaries when continuous variables were categorized	N/A
		(*c*) If relevant, consider translating estimates of relative risk into absolute risk for a meaningful time period	N/A
Other analyses	17	Report other analyses done—eg analyses of subgroups and interactions, and sensitivity analyses	10-11
**Discussion**			
Key results	18	Summarise key results with reference to study objectives	11-12
Limitations	19	Discuss limitations of the study, taking into account sources of potential bias or imprecision. Discuss both direction and magnitude of any potential bias	12-13
Interpretation	20	Give a cautious overall interpretation of results considering objectives, limitations, multiplicity of analyses, results from similar studies, and other relevant evidence	11-13
Generalisability	21	Discuss the generalisability (external validity) of the study results	13
**Other information**			
Funding	22	Give the source of funding and the role of the funders for the present study and, if applicable, for the original study on which the present article is based	17-18

*Give information separately for exposed and unexposed groups.

Note: An Explanation and Elaboration article discusses each checklist item and gives methodological background and published examples of transparent reporting. The STROBE checklist is best used in conjunction with this article (freely available on the Web sites of PLoS Medicine at http://www.plosmedicine.org/, Annals of Internal Medicine at http://www.annals.org/, and Epidemiology at http://www.epidem.com/). Information on the STROBE Initiative is available at www.strobe-statement.org.

For peer review only

How local partnerships to improve urgent and emergency care have impacted delayed transfers of care from hospitals in England: an analysis based on a synthetic control estimation method

Journal:	*BMJ Open*
Manuscript ID	bmjopen-2021-054568.R2
Article Type:	Original research
Date Submitted by the Author:	04-Jan-2022
Complete List of Authors:	Malisauskaite, Gintare; University of Kent, Personal Social Services Research Unit<br>Jones, Karen; University of Kent Personal Social Services Research Unit<br>Allan, Stephen; University of Kent, Personal Social Services Research Unit<br>Roland, Daniel; University of Kent, Personal Social Services Research Unit<br>Birks, Yvonne; University of York, Social Policy Research Unit<br>Baxter, Kate; University of York, Social Policy Research Unit<br>Gridley, Kate; University of York, Social Policy Research Unit
**Primary Subject Heading**:	Health policy
Secondary Subject Heading:	Health economics
Keywords:	HEALTH ECONOMICS, Health policy < HEALTH SERVICES ADMINISTRATION & MANAGEMENT, Organisation of health services < HEALTH SERVICES ADMINISTRATION & MANAGEMENT

SCHOLARONE™ Manuscripts

I, the Submitting Author has the right to grant and does grant on behalf of all authors of the Work (as defined in the below author licence), an exclusive licence and/or a non-exclusive licence for contributions from authors who are: i) UK Crown employees; ii) where BMJ has agreed a CC-BY licence shall apply, and/or iii) in accordance with the terms applicable for US Federal Government officers or employees acting as part of their official duties; on a worldwide, perpetual, irrevocable, royalty-free basis to BMJ Publishing Group Ltd ("BMJ") its licensees and where the relevant Journal is co-owned by BMJ to the co-owners of the Journal, to publish the Work in this journal and any other BMJ products and to exploit all rights, as set out in our licence.

The Submitting Author accepts and understands that any supply made under these terms is made by BMJ to the Submitting Author unless you are acting as an employee on behalf of your employer or a postgraduate student of an affiliated institution which is paying any applicable article publishing charge ("APC") for Open Access articles. Where the Submitting Author wishes to make the Work available on an Open Access basis (and intends to pay the relevant APC), the terms of reuse of such Open Access shall be governed by a Creative Commons licence – details of these licences and which Creative Commons licence will apply to this Work are set out in our licence referred to above.

Other than as permitted in any relevant BMJ Author's Self Archiving Policies, I confirm this Work has not been accepted for publication elsewhere, is not being considered for publication elsewhere and does not duplicate material already published. I confirm all authors consent to publication of this Work and authorise the granting of this licence.

For peer review only

How local partnerships to improve urgent and emergency care have impacted delayed transfers of care from hospitals in England: an analysis based on a synthetic control estimation method

Gintare Malisauskaite*[1], Karen Jones[1], Stephen Allan[1], Daniel Roland[1], Yvonne Birks[2], Kate Baxter[2], Kate Gridley[2]

*Correspondence: G.Malisauskaite@kent.ac.uk

Personal Social Services Research Unit (PSSRU), University of Kent, Office CC228, Cornwallis Central, Canterbury, CT2 7NF, UK

Word count: 3,446

[1] Personal Social Services Research Unit (PSSRU), School of Social Policy, Sociology and Social Research (SSPSSR), University of Kent, UK

[2] Social Policy Research Unit (SPRU), Department of Social Policy and Social Work, University of York, UK

For peer review only

Abstract

Objectives: Patients should be discharged from hospital when they are medically fit. However, discharges are often delayed for non-medical reasons including access to social care. One aim of local health and social care partnerships to improve urgent and emergency care in England (known as Urgent and Emergency Care [UEC] Vanguards) was to improve integration of health and social care, which could lead to fewer delays. Consequently, we aimed to assess the impact of UEC Vanguards on delayed discharges from hospital (Delayed Transfers of Care [DTOC]) in England.

Design: Using a synthetic control estimation method 29 local authorities (LAs) that were UEC Vanguards partners were averaged into a single 'treated' unit and compared with a unit created using data from LAs that were not UEC vanguards partners to estimate the impact of UEC Vanguards on DTOC. Sensitivity analysis included fixed effects panel regressions and various placebo tests.

Setting: 150 LAs in England (excluding city of London and Isles of Scilly); 29 LAs were partners in UEC Vanguards between August 2015 and March 2018.

Primary outcome measure: Quarterly data on days of DTOC at LA level for the period 2010 to 2017.

Results: Synthetic control estimation showed a large difference in DTOC days between UEC Vanguards partner LAs compared to those that were not, with on average 23.7% lower DTOC per quarter (491 DTOC days per quarter). Fixed effect panel regressions found DTOC rates lower by 43.1% (99% confidence interval: 13.8% to 72.4%) in UEC partner LAs after the start of the Vanguards programme. We found no indication of UEC partner LAs having lower DTOC rates prior to initiation of Vanguards.

For peer review only - http://bmjopen.bmj.com/site/about/guidelines.xhtml

For peer review only

Conclusions: The evidence indicates a sizeable statistically significant impact of UEC Vanguards on DTOC, however, more research is required to explain the underlying reasons for this relationship.

Keywords:

Delayed Transfers of Care, Urgent and Emergency Care Vanguards, integration policy

Strengths and limitations of this study:

- The long span of Delayed Transfers of Care (DTOC) data used from all English Local Authorities (LA) created a comprehensive picture of how Urgent and Emergency Care (UEC) Vanguards affected DTOC rates;
- The synthetic control method allowed for a comparison between UEC and non-UEC LAs to be drawn allowing observation of how UEC partner LAs would have been likely to perform in DTOC days in the absence of UEC Vanguards;
- The data did not permit the identification and estimation of the impact of different initiatives associated to the UEC Vanguards or account for differences in breadth and reach of UEC Vanguards within the partner LAs;
- The design did not permit a clear explanation of the mechanisms by which UEC Vanguards influenced reduction in DTOC days.

Background

Delayed Transfers of Care (DTOC) is a term used to describe situations where patients are medically fit to be discharged from hospital to home or further care settings but the process is delayed,(1, 2). DTOC has attracted increased attention from policy makers alongside health and social care professionals in England,(3), due to increased rates in recent years; there is an

For peer review only - http://bmjopen.bmj.com/site/about/guidelines.xhtml

For peer review only

estimated £820 million annual cost for the population aged 65+ (2015-2016 estimate),(4). DTOC is also associated with decreased subsequent participation in activities of daily living, frailty, aging, high comorbidity, cognitive impairment, and dependency,(5-11).

Attempts to address the costs associated with DTOC have inspired a number of innovative policy approaches to integrating social and health care, including the Better Care Fund,(12), Integrated Care Pioneers,(13) and more recently the New Models of Care – Vanguards,(14). Vanguards set out to help improve integration of services with five different approaches,(14):

- *Acute care collaboration* – linking local hospitals to improve clinical and financial viability.
- *Urgent and emergency care* – improving coordination of services and reducing pressure on A&E departments.
- *Enhanced health in care homes* – improving and integrating health, care and rehabilitation services for older people in care homes.
- *Multi-speciality community providers* – moving specialist care into the community from hospitals.
- *Integrated primary and acute care systems* – joining up GP, hospitals, community and mental health services.

This paper examines the relationship between Urgent and Emergency Care (UEC) Vanguards and DTOC rates specifically, since discharge planning from acute care in hospitals was identified as one of the challenges by the UEC Vanguards,(15). Eight UEC Vanguards were announced to take effect in July-August 2015 with a planned end date of March 2018, however a substantial number of Vanguards managed to provide enhanced services beyond that date,(16). Models of care adopted by UEC Vanguards largely aimed to better integrate the different ways that urgent and emergency care could be accessed. To facilitate service planning and design and help patients access care via the most effective and efficient routes, UEC

For peer review only - http://bmjopen.bmj.com/site/about/guidelines.xhtml

For peer review only

Vanguards were encouraged to use a tool called a Channel Shift modelling tool,(17). The tool was aimed at facilitating further integration and cooperation between health, social and community care services, and included planning for discharge from hospital from the point of admission,(14-15). Thus, DTOC was likely to be directly affected by improved discharge planning and communication between health and social care providers. In addition, DTOC rates may be a useful proxy for certain aspects of integration, and it has been previously used as a criterion for evaluating the success of health and social care integration policy initiatives,(18).

To date, research related to DTOC is somewhat scarce. Most research considers challenges related to the discharge of older people: appropriate future support, suitable discharge destinations, and how policies are put into practice,(19-22). Waiting for post-hospital care packages accounts for a large proportion of reasons for delays,(23) and lack of social care supply is considered a major part of the explanation behind DTOC,(24, 25). However, whilst delays for which social care is responsible have increased substantially, so have delays for which the NHS is deemed responsible,(26, 27). Indeed, the NHS is continuously considered responsible for the majority of delays, with internal hospital issues with planning, documentation and transport being cited as explaining increases in DTOC,(28) alongside concerns over the integration of services more generally,(29, 30). To contribute to this literature and as part of a wider study examining the role of social care in DTOC,(31) we examined the link between UEC Vanguards and DTOC rates.

Methods

Data and setting

Data were collected for 150 English Local Authorities (LAs) for the time period between 2010 quarter 4 and 2017 quarter 4 (150 LAs, 29 quarters, 4,350 observations). City of London

For peer review only - http://bmjopen.bmj.com/site/about/guidelines.xhtml

and the Isles of Scilly were excluded due to stark differences in size in comparison to other LAs. The analysis was carried out at quarterly LA level; the time frame chosen based on the availability of DTOC data at the time of analysis. The eight UEC Vanguards took effect in August 2015 (calendar year quarter 3) and included 29 Local Authorities as partners,(14). The start date of UEC Vanguards was nominal, but without more precise information on exact timings of when the programmes took effect in different locations or when it was likely to expect any impact on outcomes, we used this nominal start date as the start of the 'treatment' in our analyses.

Statistical analysis

The primary chosen method of analysis was synthetic control estimations,(32, 33). This method creates a control unit that matches the main characteristics of the treated unit so that it has a similar outcome trajectory prior to the treatment. This then allows observation of how the 'untreated' control unit would have performed in the time following the start of the treatment, providing a comparison with the treated unit. In this case the 'treatment' was participation in UEC Vanguard programme and the 'treated unit' refers to derived averages of the outcome measure and control variables,(34) of all 29 LA partners of UEC Vanguards partner sites, i.e. the 29 LAs at each quarter were used in the sample to create a single treated unit over time. The control unit was created using the remaining LAs in England (i.e. non-UEC Vanguard partners) by estimating different weights, chosen automatically by the synthetic control estimation algorithm, for LAs to account for changes in confounding variables over time as well as across LAs. Further, we controlled for the outcome variable at each quarter prior to the treatment to achieve close tracking between the two units over the period prior to the treatment. This allowed for a credible prediction of the counterfactual i.e. what would have happened in the absence of the Vanguards programme in the UEC partner LAs. DTOC trends not being

For peer review only - http://bmjopen.bmj.com/site/about/guidelines.xhtml

parallel prior to Vanguards makes synthetic control estimation a preferred method for evaluation since it does not require parallel trends assumption to hold.

The outcome measure used for synthetic control estimations was the number of DTOC days (including all delayed discharge days). The analysis controlled for the following factors associated with LA-level DTOC: (i) demographics and level of need (carer's allowance, disability living allowance, total population in LA, and percentage of population above 65 years old), (ii) LA structure (type, size in square metres, percentage of people living in rural areas, the number of Clinical Commissioning Groups (CCGs) each LA is in partnership with, and care home bed supply), and (iii) economic variables (jobseeker's allowance, pension credit, house prices, percentage of single occupancy older people home ownership. A similar set of control variables were selected by Roland et al.,(35) based on findings from the literature,(5-9,19-23) concerning confounders of delayed discharges from hospitals and data availability. Further details of the data are available in Appendix Table A1.

Sensitivity analysis

As a sensitivity check, we also estimated two-way fixed effects panel regressions (Difference-in-Difference approach). Any statistically significant results would further strengthen the argument of the existence of the relationship between UEC Vanguards and DTOC. This provided an average estimate of the effect size of the Vanguards programme on DTOC . The model specification used was:

$$DTOC_{it} = \alpha_i + V_{it}\beta_1 + V_{it}T_t\beta_2 + C_{it}\beta_3 + u_{it} \quad (1)$$

Where the dependent variable $DTOC_{it}$ is expressed as the natural logarithm of the number of DTOC days, to mitigate the potential effect of large outliers and data skewness in regression results. Subscript i indicates a Local Authority, t time (in quarters) and α is a LA dependent intercept. Vanguard partners were identified using the dummy variable V_{it} (1 = after programme start, 2015 quarter 3, 0 = before or not Vanguard partner), and β_1 is the coefficient

For peer review only - http://bmjopen.bmj.com/site/about/guidelines.xhtml

of interest for the analysis, showing the average effect of being a partner in the UEC Vanguards on DTOC. We also included interactions between participation in UEC Vanguards and time quarters $V_{it}T_t\beta_2$, with β_2 being a vector of coefficients associated with them. Finally, C_{it} is a vector of the same control variables used in the synthetic control estimation and quarter dummies for each quarter in the sample, with β_3 being a vector of coefficients associated with them, representing the effects on DTOC rate.

The equation (1) was estimated using fixed effects panel regressions,(36). A Hausman test found that a fixed effects model was preferred over a random effects model (Hausman test statistic of 46.30, significant at 1%). Some variables were dropped during the estimation process due to the invariant nature of some data (e.g. LA type and size etc.). All regressions used cluster-robust standard errors centred on LAs, to account for potential heteroscedasticity and unobservable characteristics which could make LA level clusters more similar.

Finally, given the potential for selection bias into UEC vanguards, as robustness checks we also conducted placebo tests to check if there was any evidence of significant differences in DTOC rates between UEC and non-UEC LAs by a) assuming that the UEC programme was in existence across the whole period of analysis and b) using data from prior to the start of the programme. These robustness checks were estimated using Ordinary Least Squares (OLS) and random effects (RE) given UEC vanguard partners were assumed fixed over time.

Patient and Public Involvement statement

Both public and practice stakeholders were involved in the project as part of the steering committee. They helped to shape initial study design and participated in the discussion of initial and final findings alongside dissemination. Further stakeholder engagement through dissemination at practice facing events looking at findings further shaped the presentation of early findings.

For peer review only - http://bmjopen.bmj.com/site/about/guidelines.xhtml

Results

[Table 1]

[Table 2]

Descriptive statistics

Table 1 presents descriptive statistics from the last quarter of 2010 to the last quarter of 2017. The average number of DTOC days was 2,755 per English LA per quarter. 6.7% of the sample observations were UEC Vanguard partner sites. Table 2 provides more detail on outcome and control variables based on participation in UEC Vanguard programme presenting mean values before and after the start of UEC Vanguards. Before and after means for control variables appear similar for both UEC and non-UEC partner sites, the main noteworthy difference being the sizeable increase in DTOC days after the start of the Vanguards in non-UEC LAs.

[Figure 1]

Figure 1 compares average number of DTOC days (ratio to LA population) per quarter for UEC partner LAs compared to those non-UEC LAs. The increase in DTOC days after the start of the UEC programme, obvious for the non-UEC LAs, was hardly visible for LAs in UEC Vanguards. However, since the trends prior to the Vanguards seem non-parallel, robustness checks were employed to check for any indication that selection into the UEC Vanguards programme was based on DTOC rates.

Main results

Figure 2 depicts the predictions of synthetic control estimations. The figure shows that from the beginning of the UEC Vanguards programme, partner sites had consistently lower than average DTOC rates than would have been predicted without the Vanguards. This difference was sizeable, with on average 491 fewer DTOC days per quarter per LA, or around 23.7% less (estimation outcome matrix, showing actual average days of DTOC for UEC partner LAs and

For peer review only - http://bmjopen.bmj.com/site/about/guidelines.xhtml

predicted synthetic control unit days of DTOC, is provided in Table A2 in the Appendix). The synthetic control tracking the data prior to UEC programme indicates a good fit for the model (the predictor balances between averaged treated unit and synthetic control unit are available in Table A3 in the Appendix) and standardized p-values indicate the quarterly effects are significant at 1% level (synthetic control post-estimation results in Table A4 in the Appendix),(32, 37). The sizeable dip in DTOC days just prior to the start of UEC Vanguards of, on average, 300 delayed days less per UEC Vanguard participating LA compared to the previous quarter, suggests that some form of preparation with regard to integration and DTOC could have taken place in the participating sites.

[Figure 2]

Sensitivity analysis

The first column of Table 3 reports the full results from the fixed effects panel regression estimation and shows UEC Vanguards are associated with 43.1% lower DTOC rates at 1% significance level (99% confidence interval: 13.8-72.4%), and there was no indication of any trend differences between UEC partner and non-partner LAs after the start of the programme. It was a preferred estimation for finding the coefficient size associated with UEC Vanguards impact, since when controlling for LA level characteristics Hausman test revealed systematic differences between fixed effects and random effects models and fixed effects model has an advantage of controlling for any other unobservable LA level characteristics that are fixed in time.

[Table 3]

One of the possible interpretations for the observed results may be that sites participating in UEC Vanguards could have been selected based on better DTOC rates. There was no publicly available information on specific selection criteria but testing for statistical differences between DTOC rates was carried out by running the model using two modifications:

For peer review only - http://bmjopen.bmj.com/site/about/guidelines.xhtml

a) without identifying the start of the UEC Vanguard, checking for an overall difference between UEC participating sites and others over the entire period of time of data;

b) using only data prior to 2015, the start of the initiative.

The findings (Table 3, columns a) and b) respectively) showed there was no statistically significant relationship between UEC Vanguards and DTOC in these specifications. Overall marginal effects of UEC Vanguards for these specifications were also consistently statistically insignificant. This suggests that the difference in DTOC rates between UEC and non-UEC partner LAs only becomes significant after the start of UEC programme.

Discussion

Main findings

Our findings suggest that LAs that were part of UEC Vanguards had significantly lower average DTOC than non-UEC sites after the start of the programme. Overall DTOC rates rose substantially in the second quarter of 2015 until the end of 2016, potentially explained by severe cuts to the funding of social care,(38); however, they rose significantly less, if at all, in UEC Vanguard sites. We found no evidence suggesting that UEC Vanguard sites had lower DTOC rates prior to becoming Vanguards but are unable to rule out the possibility that they might have been in a better position to reduce DTOC due to other as yet unidentified factors, e.g. other health care programmes already in place in the UEC partner LAs. It is difficult to pinpoint the reasons why UEC Vanguards influenced DTOC rates. One potential explanation from policies and initiatives associated with being in a Vanguard is the Channel Shift modelling tool-kit, which supports integration of services, communication and cooperation between hospital and community based services,(18). Channel Shift interventions include planning discharge from time of admission, discharging for further assessment ('discharge to assess')

For peer review only

and rapid response services, yet we could not formally test if this indeed is the main reason for the effect found.

There is limited previous research that has looked at the relationship between New Models of Care – Vanguards and DTOC rates. One study reported that Vanguards are associated with a small reduction in hospital admissions,(39). This highlights important implications for the National Health Service and social care, alongside individuals discharged from hospital. The move towards greater integration of services is not a new idea, and the importance of local collaboration has been consistently stressed,(40). However, it is difficult to isolate the impact of the UEC Vanguards from other Government integration policies, together with the austerity climate facing the care sector. This is particularly important with regard to potential changes the COVID-19 response has led to, including hospital discharge service, which is expected to influence the further development of discharge to assess and integration of health and social care services,(41). Although New Care Models were discontinued well before the start of the global pandemic, assumptions were made that practices adopted during the time of New Care Models activity period would continue to be used,(16). This makes it reasonable to expect this programme could have made a positive contribution towards freeing hospital beds for Covid-19 patients, however further research would be necessary to establish the size and significance of any impact. Alongside our findings, this indicates that the Vanguards programme should be of interest to policy makers in terms of lessons learned for dealing with discharges during and following the COVID-19 pandemic.

Limitations

The synthetic control estimation suggests an element of causality between UEC participation and DTOC rates. However, a number of limitations need to be acknowledged. Firstly, we could not account for selection criteria into the Vanguards, different initiatives within UEC Vanguards and the impact of other active health policies potentially influencing

For peer review only - http://bmjopen.bmj.com/site/about/guidelines.xhtml

the results during the Vanguards' activity period. Such information could further inform analysis and provide further context for this finding. Further work on specific policies used within UEC Vanguard sites, including qualitative analysis and more detailed examination of specific UEC Vanguards' mechanisms of action could help untangle potential reasons for the association between UEC Vanguards and DTOC.

Secondly, this analysis does not account for different timeframes in which UEC Vanguards took effect in different locations, differences within the eight UEC Vanguards, or possible different levels of exposure to UEC Vanguard influence in each LA. We anticipate there was some variation in associations between different UEC Vanguards and outcomes. However, this approach should be sufficient to show the average effect of the UEC Vanguards programme.

Thirdly, we did not look into re-admission rates for UEC Vanguard partner sites. Evidence suggests that there is an association between high bed occupancy and re-admission rates into hospitals in England, likely due to increased rates of discharge when bed occupancy is high,(42). There remains the possibility that hospitals in UEC partner LAs may have had higher readmission rates because of higher bed occupancy and/or improved rates of discharge. If so, the positive benefit of the UEC Vanguard programme would be mitigated somewhat. However, the UEC Vanguard programme directly addressed one of the likely causes of readmissions from high bed occupancy, i.e. inadequate discharge planning. The identified limitations could be further routes of enquiry, even if achieving precision in quantifying health care programmes is unlikely.

A potential solution to refining the analysis would be to use individual level data to conduct a similar analysis. This could allow more precise estimation of the effect size as case-specific confounders could be taken into account, which is not possible at an aggregated level.

For peer review only - http://bmjopen.bmj.com/site/about/guidelines.xhtml

For peer review only

Overall, the estimation methods adopted revealed a strong association between the UEC Vanguard and a reduction of DTOC, which advocates the success of this integration programme and should encourage further research to reveal which specific aspects of this initiative were particularly beneficial, which could guide further policy decisions.

References

1. NHS England. Delayed Transfers of Care. Leeds: NHS England; 2011 [updated 2019]. https://www.england.nhs.uk/statistics/statistical-work-areas/delayed-transfers-of-care/. Accessed Oct 2019.
2. The Kings Fund. Delayed transfers of care: a quick guide. London: The Kings Fund; 2018. https://www.kingsfund.org.uk/publications/delayed-transfers-care-quick-guide. Accessed Nov 2020.
3. NHS Providers. The rise of delayed transfers of care. London: NHS Providers; 2017. https://nhsproviders.org/the-rise-of-delayed-transfers-of-care. Accessed Oct 2019.
4. National Audit Office. Discharging older patients from hospitals. London: National Audit Office; 2016. https://www.nao.org.uk/wp-content/uploads/2015/12/Discharging-older-patients-from-hospital.pdf. Accessed Apr 2019.
5. Bo M, Fonte G, Pivaro F, et al. Prevalence of and factors associated with prolonged length of stay in older hospitalized medical patients. *GeriatrGerontolInt*2016;16(3):314-321.
6. Challis D, Hughes J, Xie C, et al. An examination of factors influencing delayed discharge of older people from hospital. *IntJGeriatrPsychiatry*2014;29(2):160-168.
7. Costa AP, Hirdes JP. Clinical characteristics and service needs of alternate-level-of-care patients waiting for long-term care in Ontario hospitals. *HealthcPolicy* 2010;6(1):32-46.
8. Costa AP, Poss JW, Pierce T, et al. Acute care inpatients with long-term delayed-discharge: Evidence from a Canadian health region. *BMCHealthServRes*2012;12:172-182.
9. McClaran J, Tover R, Duarte E. Long hospital stays and need for alternate level of care at discharge. Does family make a difference for elderly patients? *CanFamPhysician*1996;42:449-461.
10. Rojas-García A, Turner S, Pizzo E, et al. Impact and experiences of delayed discharge: A mixed-studies systematic review. *HealthExpect*2018;21(1):41-56.
11. Bai AD, Dai C, Srivastava S, et al. Risk factors, costs and complications of delayed hospital discharge from internal medicine wards at a Canadian academic medical centre: retrospective cohort study. *BMCHealthServRes*2019; doi: 10.1186/s12913-019-4760-3.
12. NHS England. Better Care Fund. London: NHS England; 2013. https://www.england.nhs.uk/ourwork/part-rel/transformation-fund/bcf-plan/. Accessed Oct 2019.

For peer review only - http://bmjopen.bmj.com/site/about/guidelines.xhtml

13. NHS England. Integrated Care Pioneers. London: NHS England; 2014. https://www.england.nhs.uk/new-care-models/integrated-care-pioneers/. Accessed Oct 2019.

14. NHS England. New Care Models: Vanguards – developing blueprint for the future of NHS and care services. London: NHS England; 2016. https://www.england.nhs.uk/wp-content/uploads/2015/11/new_care_models.pdf. Accessed Mar 2019.

15. NHS England. New Care Models: supporting the vanguards Urgent and Emergency Care (UEC) vanguards: summary. London: NHS England; 2015. https://www.england.nhs.uk/wp-content/uploads/2015/12/uec-support-package-summary.pdf. Accessed Oct 2019.

16. National Health Executive. Vanguards: Stevens pledges 10-year plan, original aims failed due to trust deficits. 2018. https://www.nationalhealthexecutive.com/Health-Care-News/vanguards-stevens-pledges-10-year-plan-original-aims-failed-due-to-trust-deficits

17. NHS England. Urgent and emergency care channel shift model. London: NHS England; 2015. https://www.england.nhs.uk/urgent-emergency-care/about-uec/uec-channel-shift/. Accessed Oct 2019.

18. Forder J, Caiels J, Harlock J, et al. A system-level evaluation of the Better Care Fund: Final report. Personal Social Services Research Unit; 2018. https://www.pssru.ac.uk/pub/5424.pdf. Accessed Oct 2019.

19. Coffey A. Discharging older people from hospital to home: what do we know? Int. *JOlderPeopleNurs*2006;1:141-150.

20. Glasby J, Littlechild R, Pryce K. All dressed up and nowhere to go? Delayed hospital discharges and older people. *JHealthServResPol*2006;11(1):52-58.

21. Swinkels A, Mitchell T. Delayed transfer from hospital to community settings: the older person's perspective. *HealthSocCareCommunity*2008;17(1):45-53.

22. Shaw J, Kontos P, Martin W, et al. The inter-relation between policy and practice for transitions from hospital to home: an ethnographic case study in England's National Health Service. *BMCHealthServRes*2014;P111. doi:10.1186/1472-6963-14-S2-P111.

23. The Nuffield Trust. Delayed transfers of care. We look at delayed transfers of care for patients following NHS treatment. 2021. The Nuffield Trust. https://www.nuffieldtrust.org.uk/resource/delayed-transfers-of-care. Accessed May 2021.

24. Gaughan J, Gravelle H, Siciliani L. Testing the bed-blocking hypothesis: does nursing and care home supply reduce delayed hospital discharges? J*HealthEcon*2015;24(1):32-44.

25. Allan S, Roland D, Malisauskaite G, et al. The influence of home care supply on delayed discharges from hospital in England. *BMCHealthServRes*2021:[forthcoming].

26. Maguire D. Delayed transfers of care: a target that misses the mark? 2018. The King's Fund. Blog. https://www.kingsfund.org.uk/blog/2018/01/delayed-transfers-care-target-misses-mark. Accessed May 2021.

27. Bottery S. Stopping the blame game around delayed transfers of care. 2017. King's Fund. Blog. https://www.kingsfund.org.uk/blog/2017/11/stopping-blame-game-delayed-transfers-care. Accessed May 2021.

For peer review only - http://bmjopen.bmj.com/site/about/guidelines.xhtml

28. Edwards N. What's behind delayed transfers of care? 2017. The Nuffield Trust. Briefing. https://www.nuffieldtrust.org.uk/resource/what-s-behind-delayed-transfers-of-care. Accessed May 2021.

29. Fernandez J, McGuire A, Raikou M. Hospital coordination and integration with social care in England: The effect on post-operative length of stay. *JHealthEcon*2018;61:233-243.

30. Goddard M, Mason A. Integrated care: A pill for all ills? *Int. JHealthPolicyManag*2017;6(1):1-3.

31. Jones K, Allan S, Malisauskaite G, et al. The influence of social care on delayed transfers of care (DTOC) among older people: Final report. Personal Social Services Research Unit. 2020. https://kar.kent.ac.uk/id/eprint/77826. [In press].

32. Abadie A, Diamond A, Hainmueller J. Synthetic Control Methods for Comparative Case Studies: Estimating the Effect of California's Tobacco Control Program. *JAmStatAssoc*2010;105(490):493-505.

33. **Abadie** A, **Diamond** A, **Hainmueller** J. Comparative Politics and the Synthetic Control Method. ***AmJPoliticalSci***2015;59(2):495-510.

34. Kreif N, Grieve R, Hangartner D, et al. Examination of the Synthetic Control Method for Evaluating Health Policies with Multiple Treated Units. *HealthEcon*2015;25(12):1514-1528.

35. Roland D, Malisauskaite G, Jones K, et al. Identifying unexplained DTOC at Local Authority level. 2020. [Forthcoming].

36. Wooldridge J M. Econometric analysis of cross section and panel data. 2nd ed. Cambridge: The MIT Press; 2010.

37. Galiani S, Quistorff B, (2017), The synth runner package: Utilities to automate synthetic control estimation using synth. *SJ*2017:17(4):834-849.

38. Bate A. Delayed transfers of care in the NHS. Briefing paper Nr. 7415, London: House of Commons Library; 2017. https://commonslibrary.parliament.uk/research-briefings/cbp-7415/. Accessed Oct 2019.

39. Morciano M, Checkland K, Billings J, et al. New integrated care models in England associated with small reduction in hospital admissions in longer-term: a difference-in-differences analysis. *HealthPolicy*2020;124(8):826-833.

40. NHS England. Why not home? Why not today? Behind every delayed transfer of care from hospital to home, there is a person, in the wrong place at the wrong time. Monthly Delayed Transfers of Care Situation Report. London: NHS England; 2018. https://www.england.nhs.uk/statistics/wp-content/uploads/sites/2/2018/11/Monthly-Delayed-Transfers-of-Care-Situation-Report-Principles-Definitions-and-Guidance.pdf. Accessed Jun 2020.

41. HM Government. Hospital Discharge Service: Policy and Operating Model. London: Department of Health and Social Care; 2020. https://assets.publishing.service.gov.uk/government/uploads/system/uploads/attachment_data/file/912199/Hospital_Discharge_Policy_1.pdf. Accessed Nov 2020.

42. Friebel R, Fisher R, Deeny SR, et al. The implications of high bed occupancy rates on readmission rates in England: A longitudinal study. *HealthPolicy*2019:123(8):765-772.

For peer review only - http://bmjopen.bmj.com/site/about/guidelines.xhtml

Abbreviations

UEC: Urgent and emergency care vanguard;

DTOC: delayed transfers of care;

LA: Local Authority;

CCG: Clinical Commissioning Group;

FE: Fixed effects panel regression model.

OLS: Ordinary least squares;

Ethics approval

Ethical approval was not required for this study due to use of publicly available secondary data. Work discussed in this article has been presented in research seminars, conferences and advisory committee meetings.

Author Contributions

GM conceived the statistical methodology, performed the statistical analysis and drafted the manuscript; KJ contributed to the conception, study design and the final writing of this paper; SA contributed to the study design, statistical methodology and the final writing of this paper; DR contributed to the statistical methodology and the final writing of this paper; YB critically revised the draft and contributed to the final writing of this paper; KB critically revised the draft and contributed to the final writing of this paper; KG critically revised the draft and contributed to the final writing of this paper.

Funding

For peer review only - http://bmjopen.bmj.com/site/about/guidelines.xhtml

This study was funded by the National Institute for Health Research School for Social Care Research, grant number C088/CM/UKJB-P116. The views expressed in this independent research publication are those of the authors and not necessarily those of the NIHR SSCR, the National Institute for Health Research or the Department of Health and Social Care.

Competing interests

The authors declare that they have no competing interests.

Data statement

Technical appendix, statistical code, and dataset available from the corresponding author on reasonable request.

[Appendix]

For peer review only

Tables and Figures

Table 1: Descriptive statistics

Variable	Mean	SD	Min/Max
**Dependent variables:**			
DTOC days	2755.394	3124.008	0/26733
DTOC(log)	7.438	0.986	0/10.194
**Explanatory variables:**			
UEC Vanguard	0.067	0.249	0/1
JSA ratio	0.026	0.016	0.001/0.09
PC ratio (65+)	0.235	0.096	0.062/0.691
CA ratio	0.011	0.004	0.004/0.027
DLA ratio (65+)	0.087	0.040	0.025/0.257
Care home beds (log)	7.553	0.825	5.451/9.461
Population (log)	12.598	0.606	10.519/14.257
Population 65+ ratio	0.166	0.043	0.06/0.286
Rurality (%)	17.507	24.468	0/100
No. CCGs to LA	5.660	3.480	1/21
House prices (£, log)	12.343	0.519	11.443/14.62
Owning single home r. (65+)	0.075	0.022	0.013/0.126
Owning single home outright r. (65+)	0.069	0.021	0.011/0.115
Area(m^2)	86839.430	150126.3	1213/803761
CCG dummy	0.655	0.475	0/1
LA type:			
Metropolitan	0.240	0.427	0/1
London	0.213	0.410	0/1
County	0.180	0.384	0/1
No of obs.		4,350	

Note: Further information on data sources and derivation of variables available in Appendix TableA1.

For peer review only - http://bmjopen.bmj.com/site/about/guidelines.xhtml

Table 2: Means of outcome and control variables by UEC Vanguard participation, before and after the start of the programme

	Non-UEC		UEC	
	Before	After	Before	After
DTOC days	2510.955	3690.847	2130.523	2233.072
DTOC(log)	7.344334	7.758407	7.255489	7.297711
JSA ratio	0.031272	0.012509	0.038485	0.016309
PC ratio (65+)	0.254087	0.190102	0.263469	0.19152
CA ratio	0.010333	0.012434	0.011437	0.014433
DLA ratio (65+)	0.087383	0.080628	0.097996	0.090038
Care home beds (log)	7.521928	7.535296	7.666286	7.666745
Population (log)	12.60945	12.63799	12.5038	12.52737
Population 65+ ratio	0.161205	0.168712	0.174282	0.183642
House prices (£, log)	12.34778	12.52908	12.03327	12.17675
Rurality (%)	16.31793		22.46746	
No. CCGs to LA	5.752066		5.275862	
Owning single home r. (65+)	0.074886		0.077415	
Owning single home outright r. (65+)	0.068836		0.071335	
Area(m^2)	83452.44		100971.3	
No of obs.	2,420	1,210	551	290

Note: Before – 2010 q4 to 2015 q2, 19 quarters; After – 2015 q3 to 2017 q4, 10 quarters.

Further information on data sources and derivation of variables available in Appendix TableA1.

For peer review only - http://bmjopen.bmj.com/site/about/guidelines.xhtml

Table 3: Regression results with DTOC (log) as dependent variable

Variable	FE	RE		OLS	
		a)	b)	a)	b)
UEC Vanguard	-0.431***	-0.0852	-0.054	-0.101	-0.053
	[0.112]	[0.150]	[0.149]	[0.149]	[0.147]
JSA ratio	-2.949	-4.046	-0.393	1.229	10.734*
	[5.746]	[4.572]	[4.330]	[4.802]	[6.065]
PC ratio (65+)	3.559	2.783**	1.478	1.229	0.832
	[2.429]	[1.414]	[1.391]	[0.848]	[0.979]
CA ratio	31.956	-4.278	-4.517	-41.223**	-60.315**
	[51.607]	[25.682]	[29.333]	[18.047]	[26.653]
DLA ratio (65+)	-1.668	-4.941**	-2.106	-0.063	1.209
	[7.182]	[2.244]	[2.498]	[1.616]	[1.756]
Care home beds (log)	-0.539	-0.401*	-0.289	-0.187	-0.264
	[0.353]	[0.213]	[0.229]	[0.161]	[0.197]
Population (log)	1.950	1.658***	1.629***	1.405***	1.540***
	[2.111]	[0.266]	[0.269]	[0.198]	[0.239]
Population 65+ ratio	16.315	12.130**	11.702**	2.336	2.641
	[10.441]	[5.602]	[5.950]	[3.246]	[4.652]
House prices (£, log)	0.028	0.107	0.161	0.070	0.195
	[0.263]	[0.146]	[0.136]	[0.133]	[0.159]
CCG dummy	0.317	0.363*	-0.034	0.715**	0.556***
	[0.419]	[0.216]	[0.123]	[0.159]	[0.149]
Rurality (%)	-	-0.009	-0.010	-0.005	-0.006
		[0.006]	[0.008]	[0.005]	[0.008]
No. CCGs to LA	-	-0.024*	-0.033**	-0.031***	-0.033**
		[0.014]	[0.015]	[0.012]	[0.014]
Home single ownership r. (65+)	-	-105.012*	-160.919***	-74.033*	-126.771**
		[56.140]	[61.456]	[40.551]	[50.798]
Home single ownership outright r. (65+)	-	104.434*	160.952***	81.398*	137.968***
		[56.140]	[60.303]	[41.406]	[50.871]
Area(m^2)	-	-3.25e-08	7.95e-09	1.86e-07	2.30e-07
		[4.76e-07]	[7.34e-07]	[5.48e-07]	[8.33e-07]
LA Type:					
Metropolitan	-	-0.308**	-0.431***	-0.202*	-0.345***
		[0.152]	[0.140]	[0.103]	[0.123]
London	-	-0.502***	-0.586***	-0.602***	-0.665***
		[0.159]	[0.162]	[0.120]	[0.142]
County	-	0.149	0.120	0.230	0.211
		[0.203]	[0.211]	[0.183]	[0.203]
Cons.	-17.234	-10.257***	-13.516***	-10.062***	-12.676***
	[28.685]	[2.839]	[2.592]	[2.181]	[2.629]
Interactions between time quarters and UEC Vanguard	Yes	Yes	Yes	Yes	Yes
Time quarter dummies	Yes	Yes	Yes	Yes	Yes
No. of obs.	4,350	4,350	2550	4,350	2550
No. of groups	150	150		150	

Note: Robust clustered standard errors in square brackets underneath the coefficients. Variables omitted during the FE estimation process due to fixed effects nature of the model: LAs' rurality (%), no. CCGs to LA, owning single home ratio (65+), owning single home outright ratio (65+), area(m^2), LA type; more details of variable construction available in the Appendix.

Robustness checks in RE and OLS columns: a) UEC Vanguard partner LAs are identified as being active with a binary variable across the whole period of analysis; b) UEC Vanguard partner LAs are identified with a binary variable only using sample prior to 2015. FE results not available for these specifications due to treatment being fixed across time in the estimations.

*** - p<1%, ** - p<5%, * - p<10%

For peer review only - http://bmjopen.bmj.com/site/about/guidelines.xhtml

Figure 1: Average DTOC days over time

[Figure 1]

Note: UEC Vanguard partner sites include 29 LA's,(14).

Figure 2: Synthetic control estimation

[Figure 2]

For peer review only

For peer review only - http://bmjopen.bmj.com/site/about/guidelines.xhtml

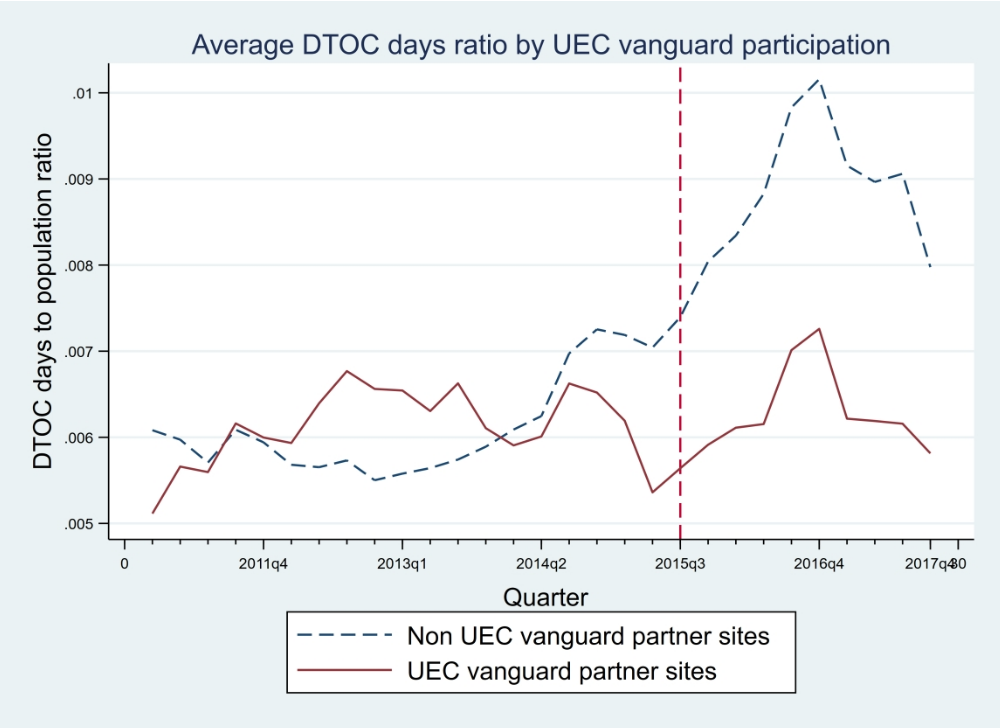

Figure 1: Average DTOC days over time

330x240mm (300 x 300 DPI)

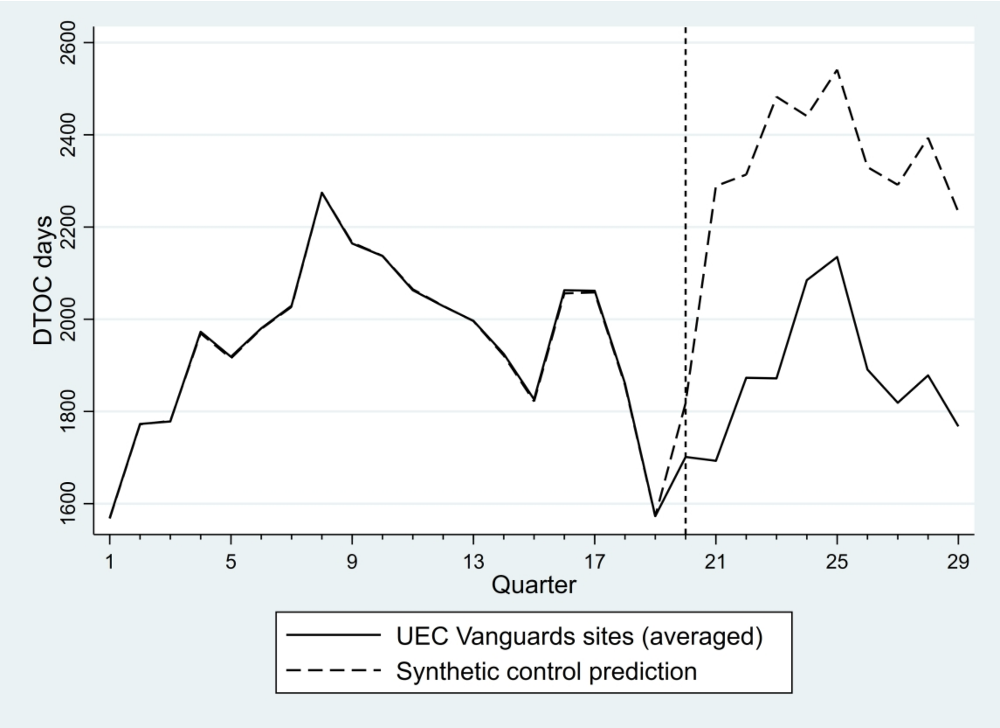

Figure 2: Synthetic control estimations

330x240mm (300 x 300 DPI)

Appendix

Table A1: Definition of variables

Variable	Explanation	Frequency	Source
**Dependent**:			
DTOC (days)	Number of days any patient experienced delayed discharge from hospital	Q	NHSE/I
DTOC (log)	Natural logarithm of DTOC(days)	Q	NHSE/I
**Explanatory**:			
UEC Vanguards	1 if one of 29 LA's participating in this Vanguard and it is onwards and after 2015 quarter 3, 0 – otherwise	F	NHSE/I
Care home beds(log)	Natural logarithm of the number of care home beds available in the LA	A	CQC
CA Ratio	Percentage of all population that receives Carer's Allowance	Q	ONS
DLA Ratio(65)	Percentage of older population (65+ years) that receives Disability Living Allowance	Q	ONS
JSA Ratio	Percentage of all population that receives Jobseeker's Allowance	Q	ONS
PC Ratio(65)	Percentage of older population (65+ years) that receives Pension Credit	Q	ONS
Population(log)	Natural logarithm of the LA's population	A	ONS
65+ ratio	Percentage of older people (65+) in the population	A	ONS
House prices(log)	Natural logarithm of average house prices in LA	Q	LandReg
Owned house	Percentage of single occupancy households of older people (65+) that own a house with mortgage, 2011 data	F	ONS
Owned house (outright)	Percentage of single occupancy households of older people (65+) that own a house outright, 2011 data	F	ONS
LA type	Type of Local Authority: Metropolitan, Unitary, London and County	F	Gov
Area	Local Authority's size (m^2)	F	Gov
Rurality	Percentage of people living in rural areas and hub towns, 2011 data	F	ONS
CCGs per LA	Number of CCGs each LA is interacting with	F	Gov
CCG dummy	1 – April 2013 onwards, identifies creation of CCG's, 0 – before April 2013	F	Gov

Note: Q – quarterly, A – annual, F – fixed; NHSE/I – NHS England and Improvement, CQC – Care Quality Commission, ONS – Office for National Statistics, LandReg – HM Land Registry's Price Paid Data, Gov – UK government's online service.

There were no missing values for all data points used throughout the chosen study time period.

For peer review only

Table A2: Synthetic control estimation outcome (DTOC) measures by period:

Period	DTOC days Treated	DTOC days Control (synth)	Difference	Difference in % (to Treated)
1	1569.36	1567.916	-1.444	-0.09201
2	1772.96	1772.207	-0.753	-0.04247
3	1778.4	1779.567	1.167	0.065621
4	1972.96	1969.42	-3.54	-0.17943
5	1918.64	1915.392	-3.248	-0.16929
6	1980.32	1979.21	-1.11	-0.05605
7	2029.24	2026.926	-2.314	-0.11403
8	2274.52	2274.597	0.077	0.003385
9	2164.28	2166.296	2.016	0.093149
10	2137.16	2137.553	0.393	0.018389
11	2062.92	2065.035	2.115	0.102525
12	2028.56	2029.041	0.481	0.023711
13	1996.64	1996.134	-0.506	-0.02534
14	1924.96	1921.385	-3.575	-0.18572
15	1826.32	1821.821	-4.499	-0.24634
16	2063	2056.206	-6.794	-0.32933
17	2061.84	2058.22	-3.62	-0.17557
18	1860.92	1857.615	-3.305	-0.1776
19	1573.04	1572.29	-0.75	-0.04768
**20**	**1701.48**	**1818.133**	**116.653**	**6.855972**
**21**	**1693.04**	**2288.897**	**595.857**	**35.1945**
**22**	**1873.08**	**2313.684**	**440.604**	**23.52297**
**23**	**1871.88**	**2482.475**	**610.595**	**32.61935**
**24**	**2084.72**	**2440.76**	**356.04**	**17.07855**
**25**	**2135**	**2541.185**	**406.185**	**19.02506**
**26**	**1890.96**	**2330.016**	**439.056**	**23.21868**
**27**	**1819**	**2291.485**	**472.485**	**25.97499**
**28**	**1878.28**	**2393.571**	**515.291**	**27.4342**
**29**	**1767.72**	**2232.34**	**464.62**	**26.28357**

Note: Nominal UEC Vanguards start date was 3rd quarter of 2015, quarter 20 in our dataset, results after the start of this Vanguard indicated in bold.

For peer review only

Table A3: Synthetic control estimation predictor balance:

Variable	Treated	Synthetic
JSA ratio	0.039029	0.040455
PC ratio (65+)	0.269154	0.28076
CA ratio	0.011704	0.012353
DLA ratio (65+)	0.098542	0.103666
Care home beds (log)	7.602731	7.35620
Population (log)	12.43287	12.35283
Population 65+ ratio	0.173527	0.160181
Rural or hub (%)	20.8956	12.78465
No. CCGs to LA	4.84	3.924
House prices (£, log)	12.03679	11.99105
Owning single home r. (65+)	0.077565	0.07411
Owning single home outright r. (65+)	0.071337	0.068227
Area(m^2)	79959.32	76051.94

Table A4: Synthetic control post-estimation results:

Post estimation period	Estimates (synth-treated)	p-vals	p-vals standardized
c1 (quarter 20)	-116.653	0.77686	0
c2 (quarter 21)	-595.857	0.38843	0
c3 (quarter 22)	-440.604	0.545455	0
c4 (quarter 23)	-610.595	0.446281	0
c5 (quarter 24)	-356.04	0.719008	0
c6 (quarter 25)	-406.185	0.710744	0
c7 (quarter 26)	-439.056	0.677686	0
c8 (quarter 27)	-472.485	0.628099	0
c9 (quarter 28)	-515.291	0.603306	0
c10 (quarter 29)	-464.62	0.603306	0

Note: post-estimation results using synth_runner command on Stata.

For peer review only

STROBE Statement—Checklist of items that should be included in reports of ***cross-sectional studies***

	Item No	Recommendation	Page No
**Title and abstract**	1	(*a*) Indicate the study's design with a commonly used term in the title or the abstract	1
		(*b*) Provide in the abstract an informative and balanced summary of what was done and what was found	2
**Introduction**			
Background/rationale	2	Explain the scientific background and rationale for the investigation being reported	4-5
Objectives	3	State specific objectives, including any prespecified hypotheses	5
**Methods**			
Study design	4	Present key elements of study design early in the paper	2, 6
Setting	5	Describe the setting, locations, and relevant dates, including periods of recruitment, exposure, follow-up, and data collection	5-6
Participants	6	(*a*) Give the eligibility criteria, and the sources and methods of selection of participants	N/A
Variables	7	Clearly define all outcomes, exposures, predictors, potential confounders, and effect modifiers. Give diagnostic criteria, if applicable	6-8
Data sources/ measurement	8*	For each variable of interest, give sources of data and details of methods of assessment (measurement). Describe comparability of assessment methods if there is more than one group	Appendix A1
Bias	9	Describe any efforts to address potential sources of bias	7-8, 12-13
Study size	10	Explain how the study size was arrived at	5-6
Quantitative variables	11	Explain how quantitative variables were handled in the analyses. If applicable, describe which groupings were chosen and why	7-8
Statistical methods	12	(*a*) Describe all statistical methods, including those used to control for confounding	6-8
		(*b*) Describe any methods used to examine subgroups and interactions	N/A
		(*c*) Explain how missing data were addressed	N/A
		(*d*) If applicable, describe analytical methods taking account of sampling strategy	6
		(*e*) Describe any sensitivity analyses	7-8
**Results**			
Participants	13*	(a) Report numbers of individuals at each stage of study—eg numbers potentially eligible, examined for eligibility, confirmed eligible, included in the study, completing follow-up, and analysed	N/A
		(b) Give reasons for non-participation at each stage	N/A
		(c) Consider use of a flow diagram	N/A
Descriptive data	14*	(a) Give characteristics of study participants (eg demographic, clinical, social) and information on exposures and potential confounders	9
		(b) Indicate number of participants with missing data for each variable of interest	N/A
Outcome data	15*	Report numbers of outcome events or summary measures	9

For peer review only

For peer review only - http://bmjopen.bmj.com/site/about/guidelines.xhtml

Main results	16	(*a*) Give unadjusted estimates and, if applicable, confounder-adjusted estimates and their precision (eg, 95% confidence interval). Make clear which confounders were adjusted for and why they were included	9-10
		(*b*) Report category boundaries when continuous variables were categorized	N/A
		(*c*) If relevant, consider translating estimates of relative risk into absolute risk for a meaningful time period	N/A
Other analyses	17	Report other analyses done—eg analyses of subgroups and interactions, and sensitivity analyses	10-11
**Discussion**			
Key results	18	Summarise key results with reference to study objectives	11-12
Limitations	19	Discuss limitations of the study, taking into account sources of potential bias or imprecision. Discuss both direction and magnitude of any potential bias	12-13
Interpretation	20	Give a cautious overall interpretation of results considering objectives, limitations, multiplicity of analyses, results from similar studies, and other relevant evidence	11-13
Generalisability	21	Discuss the generalisability (external validity) of the study results	13
**Other information**			
Funding	22	Give the source of funding and the role of the funders for the present study and, if applicable, for the original study on which the present article is based	17-18

*Give information separately for exposed and unexposed groups.

Note: An Explanation and Elaboration article discusses each checklist item and gives methodological background and published examples of transparent reporting. The STROBE checklist is best used in conjunction with this article (freely available on the Web sites of PLoS Medicine at http://www.plosmedicine.org/, Annals of Internal Medicine at http://www.annals.org/, and Epidemiology at http://www.epidem.com/). Information on the STROBE Initiative is available at www.strobe-statement.org.

For peer review only
